# Benchmarking Zinc-Binding Site Predictors: A Comparative Analysis of Structure-Based Approaches

**DOI:** 10.1021/acs.jcim.5c00549

**Published:** 2025-05-15

**Authors:** Cosimo Ciofalo, Vincenzo Laveglia, Claudia Andreini, Antonio Rosato

**Affiliations:** † Department of Chemistry, 9300 University of Florence, Via della Lastruccia 3, Sesto Fiorentino 50019, Italy; ‡ Magnetic Resonance Center (CERM), University of Florence, Via Luigi Sacconi 6, Sesto Fiorentino 50019, Italy; § Consorzio Interuniversitario di Risonanze Magnetiche di Metallo Proteine, Via Luigi Sacconi 6, Sesto Fiorentino 50019, Italy

## Abstract

Metalloproteins play crucial physiological roles across all domains of life, relying on metal ions for structural stability and catalytic activity. In recent years, computational approaches have emerged as powerful and increasingly reliable tools for predicting metal-binding sites in metalloproteins, enabling their application in the challenging field of metalloproteomics. Given the growing number of available tools, it is timely to design a reproducible approach to characterize their performance in specific usage scenarios. Thus, in this study, we selected some state-of-the-art structure-based predictors for zinc-binding sites and evaluated their performance on two data sets: experimental apoprotein structures and structural models generated by AlphaFold. Our results indicate that apoprotein structures pose significant challenges for predicting metal-binding sites. For these systems, the predictors achieved lower-than-expected performance due to the structural rearrangements occurring upon metalation. Conversely, predictions based on AlphaFold models yielded significantly better results, suggesting that they more closely resemble the holo forms of metalloproteins. Our findings highlight the great potential of metal-binding site predictions for advancing research in the field of metalloproteomics.

## Graphical abstract

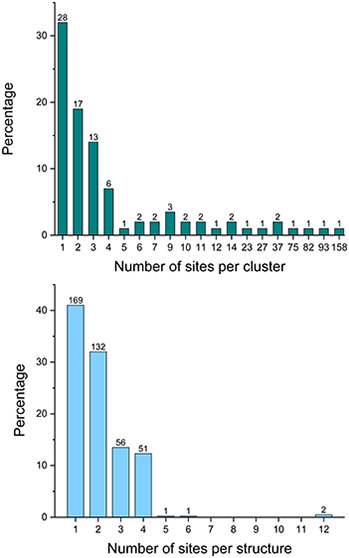

## Introduction

Metalloproteins are a varied group of proteins that harbor metal ions as crucial structural components.^1^ They are present in all forms of life and play a variety of physiological roles such as catalysis, electron transfer, oxygen transport, and gene regulation.^2^ Metal ions can serve multiple functions in metalloproteins.^3^ They can be structural elements, stabilizing the protein, or catalytic components, activating substrates or stabilizing reaction intermediates. Metal ions can also transport electrons between redox-active sites, regulate protein activity, and transmit cellular signals.

Metalloproteomics is the study of metalloproteins at the whole organism or whole cell level.^4^ Due to the difficulties of experimental metalloproteomics, bioinformatics has emerged as an alternative method for mining metalloproteomes.^5^ In this context, 3D structure-based prediction of the presence of metal-binding sites, leveraging knowledge about the relative location in space of amino acids that may provide donor atoms for metal coordination, is an area of application that has received a lot of attention.^6^ The success of AlphaFold^7^ in the CASP programs^8^ has given a significant boost to such approaches, thanks to the extensive availability of viable 3D structural models for proteins not yet described experimentally. Notably, in 2024 the release of version 3 of AlphaFold included the possibility to predict the binding sites for some metal ions and metal-containing cofactors.^9^ Such predictors leverage the information on metal-binding sites extracted from the PDB. In principle, it would be relevant to ascertain whether any PDB site used for training is physiologically relevant or is an artifact due to experimental conditions, such as high metal concentration in the crystallization buffer. Previous analyses have shown that nonphysiological sites tend to occur at the protein surface and to have metal coordination numbers lower than physiological sites.^10–12^ The number of protein residues in the second coordination sphere is also a useful indicator.^11^ It is therefore possible to train predictors using data sets that have been cleansed of a significant share of nonphysiological sites; for the present work it is also relevant that the MetalPDB database provides extensive annotation regarding the physiological relevance of zinc sites.^13^ This has been indeed implemented in some 3D structure-based predictors of such sites.^14,15^

There are currently many structure-based predictors of metalloproteins available,^14–24^ with reported performance ranging from good to excellent. It is thus difficult for the user to determine which tool(s) are best suited to their needs. Consequently, we decided to benchmark some of the most recently published tools and use the results to provide an overview of the expected outcomes in typical application scenarios. To this end, we designed two different benchmarks. The first is based on the experimental structures of metalloproteins that lack their metal cofactor(s) (so-called apo-proteins). The second benchmark takes advantage of the high-quality structural models from the AlphaFold database.^25^ We focused on zinc-binding proteins, as most predictors have been trained on this subgroup of metalloproteins owing to their abundance in the Protein Data Bank. Our findings indicate that apoprotein structures are somewhat difficult for the predictors, who performed below expectations. When we used input structures from the AlphaFold database, however, we obtained excellent results for some predictors. This suggests that the identification of zinc metalloproteomes is indeed achievable with currently available tools.

## Methods

The methods section is divided into two parts to reflect the two distinct workflows employed in this study. The first part describes the benchmarking of state-of-the-art zinc-protein (ZnP hereafter) predictors using a data set of apo-proteins derived from MetalPDB.^13,26^ The second part details the application of these predictors, along with AlphaFold 3,^9^ for the prediction of zinc-binding sites in the Saccharomyces cerevisiae proteome.

### Benchmarking of ZnP Predictors Based on Apo-Proteins

#### Data Set Creation

The predictors were tested using the apo structures of metalloproteins as input. An apoprotein is a protein that lacks its prosthetic group, such as a metal cofactor, while an holoprotein is a protein bound to its prosthetic group. All the data for constructing the data set for the benchmark was obtained from MetalPDB. This database groups collections of 3D templates with the same metal-binding site (MBS hereafter) into clusters. The MBS is defined by including any residue or chemical species having at least one atom within 5.0 Å of a metal ligand, where a metal ligand is a residue harboring any non-hydrogen atom within 3.0 Å from the metal. These clusters contain “equistructural” and “equivalent” sites. Two MBSs are defined as equivalent in MetalPDB if they satisfy all the following conditions: (i) they are found in PDB chains with the same structure (based on Pfam domain composition or on the sequence identity between the two chains being ≥50%); (ii) after structural superposition of the PDB chains, they are superimposed with the metal atoms in the same position; (iii) they contain the same metal(s). Condition (iii) is lifted in the definition of equistructural sites, consequently two equivalent sites are also equistructural but the vice versa may not be true, as two equistructural sites can harbor different metals.

We built the initial data set by including only clusters of equivalent mononuclear zinc sites (CLES) and their corresponding apo structures. From these clusters, we selected those that were physiologically relevant and contained only aspartate, cysteine, glutamate, and histidine as the metal-binding residues. The latter are the most common residues in zinc sites^26–28^ The selection of physiological sites followed the criteria outlined by Laveglia et al.^11^

The final data set for the benchmark included 87 CLES, containing 412 apo structures, for a total of 840 sites. The number of sites is greater than the number of apo structures because an apo structure can have multiple sites, with each site possibly belonging to a different CLES. This information is provided as Supporting Table S1.

#### Predictors Used and Calculation Setup

We used several state-of-the-art metalloprotein predictors, such as BioMetAll,^18^ Metal1D (M1D),^15^ Metal3D (M3D),^15^ GASS,^21^ and Master of Metals (MoM).^14^ The latter was developed in our lab. Each of these predictors was selected based on their demonstrated effectiveness in previous studies and their ability to handle the specific requirements of ZnP prediction. All are structure-based predictors, using homology to known structures or distance features to infer the location of metals. BioMetAll is a geometrical predictor that identifies MBSs based on backbone preorganization, it operates under the assumption that the geometric patterns of a protein’s backbone encode sufficient information about its preorganization to coordinate metal ions. M1D is a distance-based predictor that infers the location of MBSs by utilizing coordination motifs extracted from the Protein Data Bank.^29^ M3D is a deep learning-based predictor that operates on a voxelized representation of the protein environment and predicts metal density on a per-residue basis. GASS uses a parallel genetic algorithm to find candidate MBSs that are structurally similar to curated templates from the M-CSA^30^ and MetalPDB ^26^databases. MoM employs a neural network followed by a filter that compares the network output against the local structures of all known sites. This filter operates by comparing the distance matrices of the Cα and Cβ atoms within the sites.

The parameters of each metalloprotein predictor were set as specified in their respective original articles to ensure the best possible output quality. For each predictor, we selected the top five outputs for every input based on its own ranking criteria. If the authors suggested a threshold for the score associated with their predictor’s output, we discarded any predictions falling below that threshold. As a result, in some cases, less than five predictions were selected. This is further detailed below.

BioMetAll predicts MBSs by embedding a protein in a grid of fictitious metal probes, which are evaluated using geometric features. For each probe, the program identifies nearby amino acids that meet a set of predefined geometric criteria. For each output, we select the 5 predictions associated with the highest number of probes.

M1D scans all residues in the protein for compatibility with a computed probability map. Metals are positioned at the geometric center of residues that have high scores according to the map. The final ranking of the potential MBSs is then established using these probability scores. Thus, we selected the 5 predictions associated with the highest number of probability scores.

M3D processes a protein structure and a set of residues as input, converting the environment around each residue into a voxelized format. It then predicts the metal density for each residue individually. These per-residue predictions are averaged to produce an overall zinc density (ρ) for the entire protein. We retain the best five outputs with a ρ greater than or equal to 0.75.

GASS identifies MBS on protein structures using a parallel genetic algorithm and templates from the M-CSA and MetalPDB databases. GASS evaluates the effectiveness of potential binding sites using a fitness value, which measures the match between the candidate sites and the reference templates based on structural data. We selected the 5 predictions associated with the lowest value of fitness, where a lower fitness indicates a better result.

MoM identifies triads or quadruplets of amino acids with appropriate relative spatial arrangements from the PDB input using a machine learning algorithm. These identified structures are then ranked based on their structural similarity to a library of templates extracted from the MetalPDB database. The top 5 outputs with the best similarity scores values were selected.

#### Benchmarking Procedure

The benchmarking procedure involved using the apo structures as input and the corresponding holo structures to determine the accuracy of the MBS predictions.

All procedures and data manipulation were conducted using homemade Python scripts. The jobs for all predictors were launched on the local cluster. M1D and M3D predict the binding location, so their output consists of the coordinates of the metal ion itself. In contrast, BioMetAll, MoM and GASS are binding site predictors and their output indicates the residues that bind the ion. In this benchmark, we used as the criterion for the validation of the output the ability to correctly predict the residues binding to the metal ion (so-called metal ligands). Therefore, to validate the outputs of M1D and M3D, we considered the residues within 3.8 Å of the metal position. This distance was chosen based on the statistics available for zinc sites in MetalPDB. The prediction of an individual metal site composed of n residues was considered a true positive (TP) if at least n-1 residues were correctly predicted; otherwise, it was considered a false positive (FP).

We conducted a second type of analysis to assess the capability of the predictors to globally predict a cluster of structures (i.e., a CLES). In this context, a CLES was considered identified if at least 50% of the sites within it were correctly predicted. This type of analysis aims to determine the sensitivity of the predictors to the rearrangement of side chains that can occur in apo sites. As extensively reported,^31^ donor atoms can experience significant changes in their relative positions when the metal is absent. To quantify how much this phenomenon is present in each CLES we defined a donor atom or donor atom proxy for each residue of the CHED group: for aspartate, we chose the Cγ atom; for cysteine, Sγ; for glutamate, Cδ; and for histidine, Cε1. Once the ″donors″ were defined, we calculated the distance matrix for each apo site in a CLES using the distances between the donor atoms. All matrices are symmetric with zeros on the diagonal; for the same CLES, they all have the same *n* × *n* dimension, where *n* is the number of ligands in the site. These matrices were organized into a tensor of dimensions *N* × *n* × *n*, where *N* is the number of apo sites in the CLES. Considering only the upper part of the tensor, we calculated the standard deviation for each position on the vector along the N dimension. Then, by taking all the vectors for every position, we created a violin plot where each violin represents the distribution of distances between two donors within the CLES. Subsequently, we explored whether significant rearrangements of the side chains of individual residues could co-occur with similar shifts in other residues within the same site. For this investigation, we selected matrices from the tensor that exhibited at least two elements with values exceeding two standard deviations above the difference from the mean matrix. We then analyzed these identified sites to find connections between the side chain movements of residues within each site.

### Evaluation of the Best Predictors on the S. cerevisiae Proteome

#### Data Set Creation

In this benchmark, we selected proteins from the S. cerevisiae proteome that do not have homologous metalloproteins with a deposited 3D structure in the PDB. To identify homologs, the BLAST software was used with an E-value threshold of 0.01.^32^ This approach allowed us to evaluate the performance of the algorithms, considering the structural diversity expected across a whole proteome. The selection of proteins without a structurally characterized homolog reduced the risk of introducing bias and provided a greater challenge in the prediction process, because all the predicted MBSs were in principle unprecedented sites. All the input structures were obtained from the AlphaFold 2 database,^25^ with at least 90% of their residues having a pLDDT > 0.7. Following the procedures outlined in our previous work,^14^ we further filtered the data set, resulting in 67 structures used to test whether the selected predictors agreed with each other in the identification of novel zinc-proteins. The UniProt identifiers of these proteins are given in Supporting Table S2.

#### Predictors Used

In this second part of the work, MoM, M3D, and GASS, the three predictors that performed best in the first part of the benchmark, were used with the same settings already described, alongside AlphaFold 3 (AF3).^9^ AF3 is a deep-learning model designed to predict the 3D structure of biomolecular complexes, including proteins, nucleic acids, ligands, and metal ions. The results of AF3 were obtained using the proprietary web server.

#### Benchmarking Procedure

Given the lack of a reference holo structure to evaluate the predictions, the following approach was used: a protein was considered a ZnP if at least two predictors identified the same site. Notably, AF3 requires the identity and number of metals to predict as an input parameter. Since the number of requested sites can alter the protein structure output by AF3, the initial number of sites for each structure was set to 1. If the number of identified sites, based also on the output of the other three predictors, was greater than one, the AF3 prediction was repeated using as input the total number of predicted sites. The combined number of sites identified by all predictors in a single protein was three at most. For the construction of the confusion matrix, outputs from AF3 with a pLDDT < 0.7 for the predicted MBS, M3D with a ρ score <0.75, and MoM with a d_min >0.35 were considered and classified as negative predictions, further categorized as either true negatives or false negatives. Conversely, outputs related to the identified sites in the input structure, ranked among the top five predictions from each predictor, were classified as true positives or false positives.

## Results

### Description of the Benchmark

The first benchmarking procedure involved using apo structures as input and their corresponding holo structures to evaluate the accuracy of metal site predictions. The predictors used were BioMetAll, GASS,^21^ M1D,^15^ M3D,^15^ and MoM.^18^ The data set comprises 412 structures containing 840 zinc­(II) sites in their apo form, organized into 87 clusters (CLESs). The number of sites exceeds the number of apo structures because a single structure can include multiple sites, potentially belonging to different CLESs. The methodology used to construct the clusters is detailed in the Methods Section. Figure 1 shows the distribution of the number of sites across the structures and the distribution of the number of sites within the CLESs. 73% of the apo structures in the data set contain either one or two sites; 48% percent of the total sites belong to the four largest CLESs.

**Figure 1:**
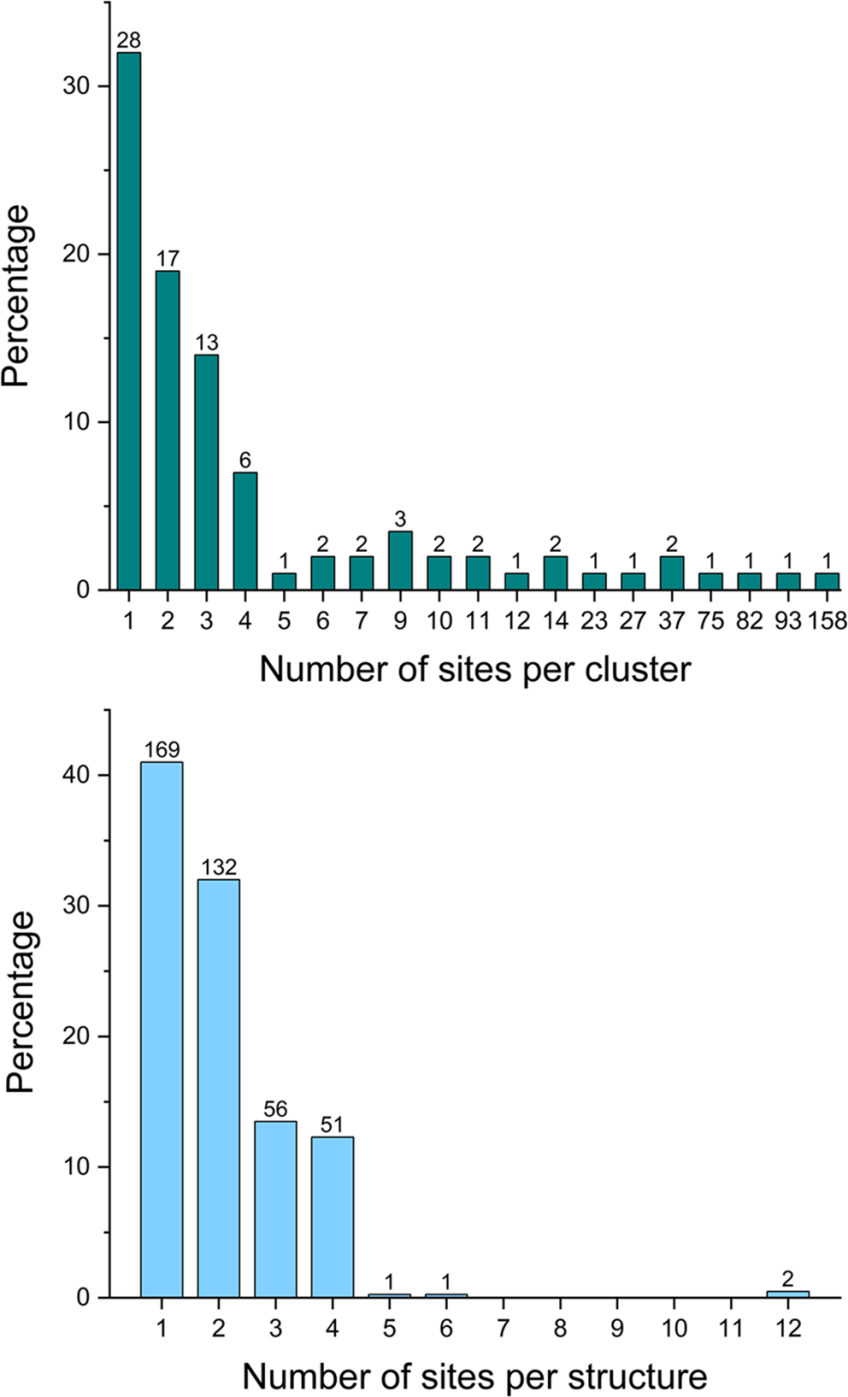
Percentage distribution of sites across CLESs and structures. (Top) The histogram shows the percentage distribution of the number of sites across CLESs. The number above each bar indicates how many CLESs have the corresponding number of sites. (Bottom) The histogram shows the percentage distribution of the number of sites per structure. The number above each bar indicates how many structures harbor the corresponding number of sites.

Given the use of experimental apo structures, a qualitative investigation was conducted to assess the potential spread of distances between donor atoms of the side chains in equivalent sites across different structures. To visualize this, we generated a violin plot for each CLES. The graphical results indicate that this phenomenon is present in many CLESs, generally becoming more pronounced as the number of sites within the CLES increases. The four largest CLESs show the most appreciable spread. We then explored whether significant rearrangements of the side chains of individual residues were associated with shifts in other residues within the same site. Our analysis revealed a notable co-occurrence between the shifts of the side chains within a site, suggesting that the rearrangement of one residue often aligns with coordinated movements of other residues in the same binding site. For example, in a site composed of three residues, if one residue deviates significantly from the average distance, it naturally causes two distances to fall outside the average, however, we frequently observed that all three residues deviate together, resulting in all distances being outside the average.

Details on how the values for the plots were calculated are provided in the Methods Section. Figure 2 shows the violin plot for a CLES composed of 31 sites with a Cys-Asp-His binding pattern. The corresponding plots for all CLESs are provided as Supporting Figures S1–S15.

**Figure 2:**
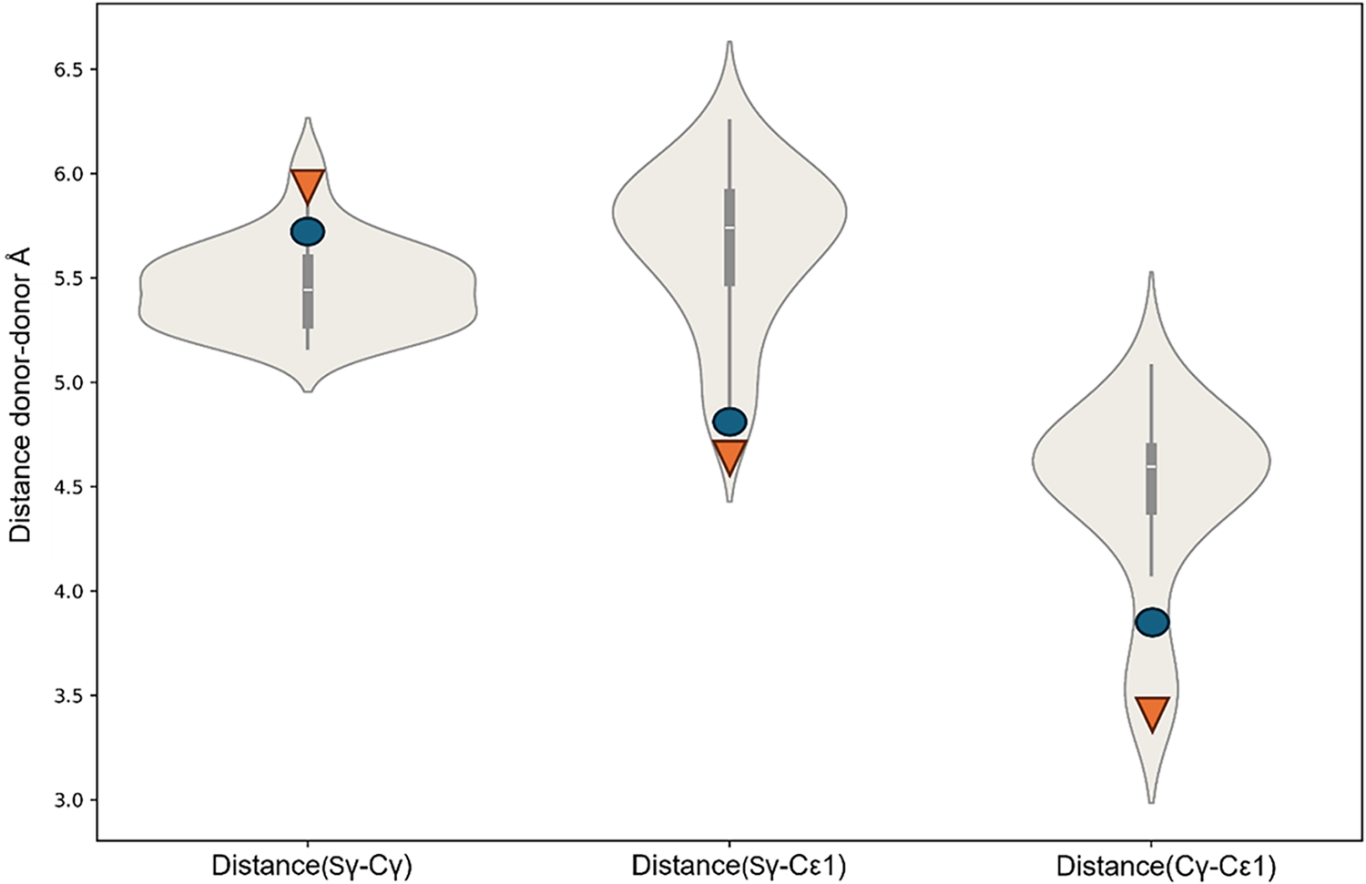
Violin plot for CLES 22022, composed of 31 sites. Each violin represents the distribution of the distance between two selected donor atoms in the binding site. The white line at the center of each violin indicates the median value, while the bars around it mark the first and third quartiles. The circles and the triangles of the same color correspond to the sites whose distance matrices contain at least two elements with values beyond two standard deviations from the mean. the site of this cluster consists of 1 cysteine (Sγ), 1 aspartate (Cγ) and 1 histidine (Cε1), where Sγ, Cγ and Cε1 are the donor atom or donor atom proxy.

### Evaluation of the Predictions

Table 1 presents the results of the benchmark based on the number of predictions considered for each predictor for each input structure. Each predictor was evaluated based on its top 1, top 3, top 5, and top 10 outputs. The number of sites used to compute the recall was determined by the number of available binding sites in each structure. For instance, for the evaluation of the top 1 outputs, only one site per structure was considered; for the top 3, a maximum of three sites were considered in structures with at least that many known sites, and so on. Hereafter, we will describe the results obtained by considering the top 5 outputs from each predictor for every input apo structure, this approach provides the optimal balance between the total number of sites and the number of predictions evaluated for each structure.

**Table 1:** Benchmark Results for Each Predictor^a^

evaluated predictions	predictor	N. outputs	N. true positives	N. redundant	N. false positives	precision (%)	recall (%)
top 1	M1D	385	91	0	294	23.6	21.6
	BioMetAll	338	52	0	286	15.4	12.1
	GASS	411	143	0	268	34.8	34.5
	MoM	238	221	0	17	92.8	53.4
	M3D	239	154	0	85	64.4	36.9
top 3	M1D	759	126	2	633	16.6	16.2
	BioMetAll	1013	159	57	854	15.7	13.3
	GASS	1206	416	106	790	34.5	40.5
	MoM	521	467	134	54	89.6	43.5
	M3D	455	260	26	195	57.1	30.5
top 5	M1D	844	129	2	715	15.3	15.4
	BioMetAll	1687	260	116	1427	15.4	17.5
	GASS	1981	626	236	1355	31.6	47.3
	MoM	693	609	237	84	87.9	45.1
	M3D	503	272	27	231	54.1	29.7
top 10	M1D	854	129	2	725	15.1	15.2
	BioMetAll	4030	654	381	3376	16.2	32.7
	GASS	3897	1061	576	2836	27.2	58.0
	MoM	868	726	331	142	83.6	47.2
	M3D	527	279	28	248	52.9	30.0

a The columns in the table, from left to right, report the number of predictions included in the analysis for each predictor, the name of the predictor used (Predictor), the total number of outputs generated by the predictor for the benchmark (N. Outputs), the number of correct predictions (N. True Positives), the number of repeated TP predictions for the same site (N. Redundant), the number of incorrect predictions (N. False Positives), the percentage of TPs over the total predictions including redundant sites (Precision %), and the percentage of TPs over the total known binding sites for that structure (Recall %)

Before going into the detailed analysis of the performance of the tools, it is important to point out that only GASS consistently provided an output for 411 input structures, with only input structure causing the program to crash. At the other extreme, M3D and MoM provided an output for only 239 and 238 structures, respectively, corresponding to 54% of the data set. This is due to either all the candidate sites following below the set thresholds or, in a limited number of instances, the programs crashing. Since the GASS authors did not specifically suggest a threshold for its outputs, we gathered all of them. Figure 3 shows the percentages of true positives (TP), redundant true positives (RTP), false positives (FP) and the Recall for all predictors. The percentages of TP, RTP, and FP are calculated with respect to the total number of site predictions, while the Recall is based on the total number of sites in the data set. BioMetAll and M1D exhibit a high number of false positives, and they provide correct predictions for less than 20% of the sites in the database. GASS, M3D and MoM generally achieve better results. GASS achieves the best result in terms of Recall, due to its ability to produce outputs for all input structures as noted above, but generates many more false positives than M3D and MoM. The latter two predictors are generally more balanced than GASS. Among them, MoM achieves the highest percentage of true positives, even when excluding the RTP. Thus, when looking at all output predictions, MoM and M3D have comparable precision, between 50% and 60%, somewhat better than the 30% achieved by GASS.

**Figure 3:**
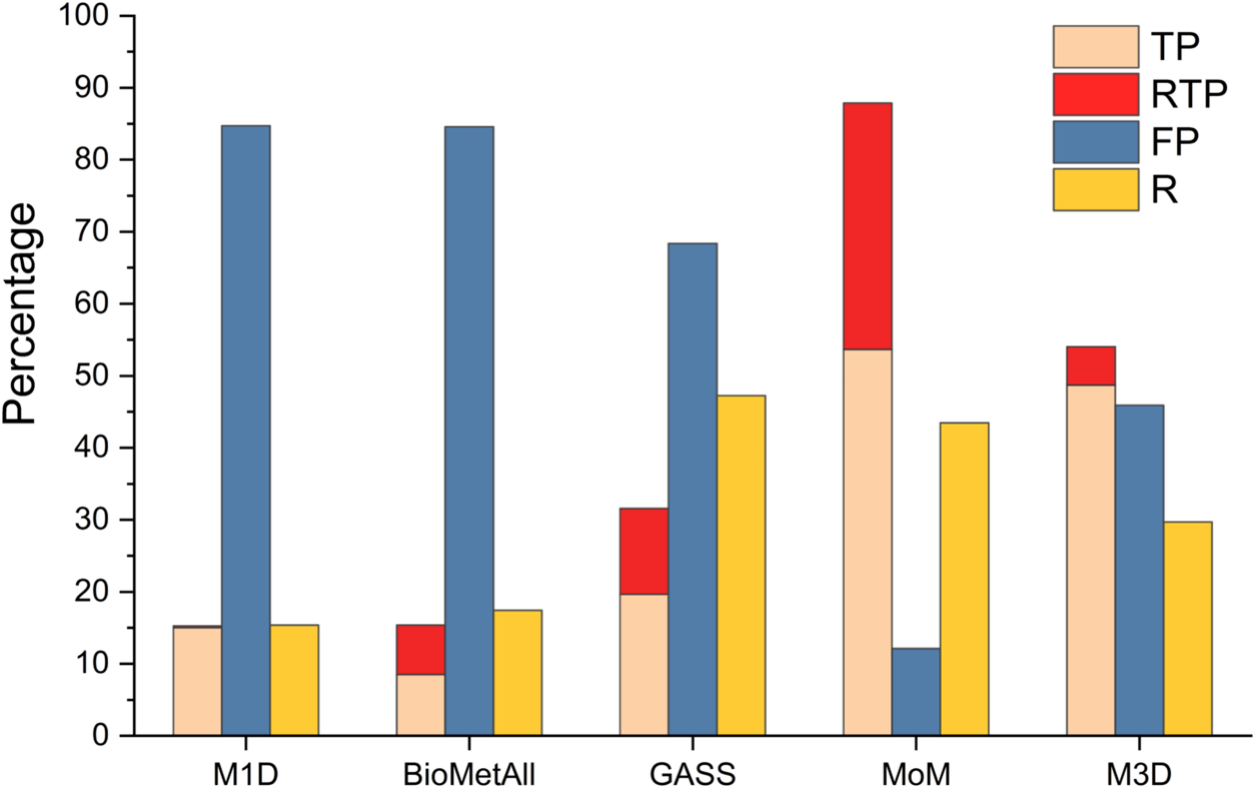
Performance of the predictors, based on the top 5 outputs. True positive (TP), redundant true positive (RTP), false positive (FP) and recall (*R*) values are displayed for all the predictors.

In order to understand the results of the tools at the level of protein families rather than individual MBS, Figure 4 shows the recall of the predictors across all CLESs. GASS, M3D, and MoM are the top performers. GASS achieves the best results overall (59% of CLESs identified), which may be due to the high number of predictions it generates compared to the other two predictors. Importantly, the recall of MoM is relatively close (54%), owing to its significantly higher precision.

**Figure 4:**
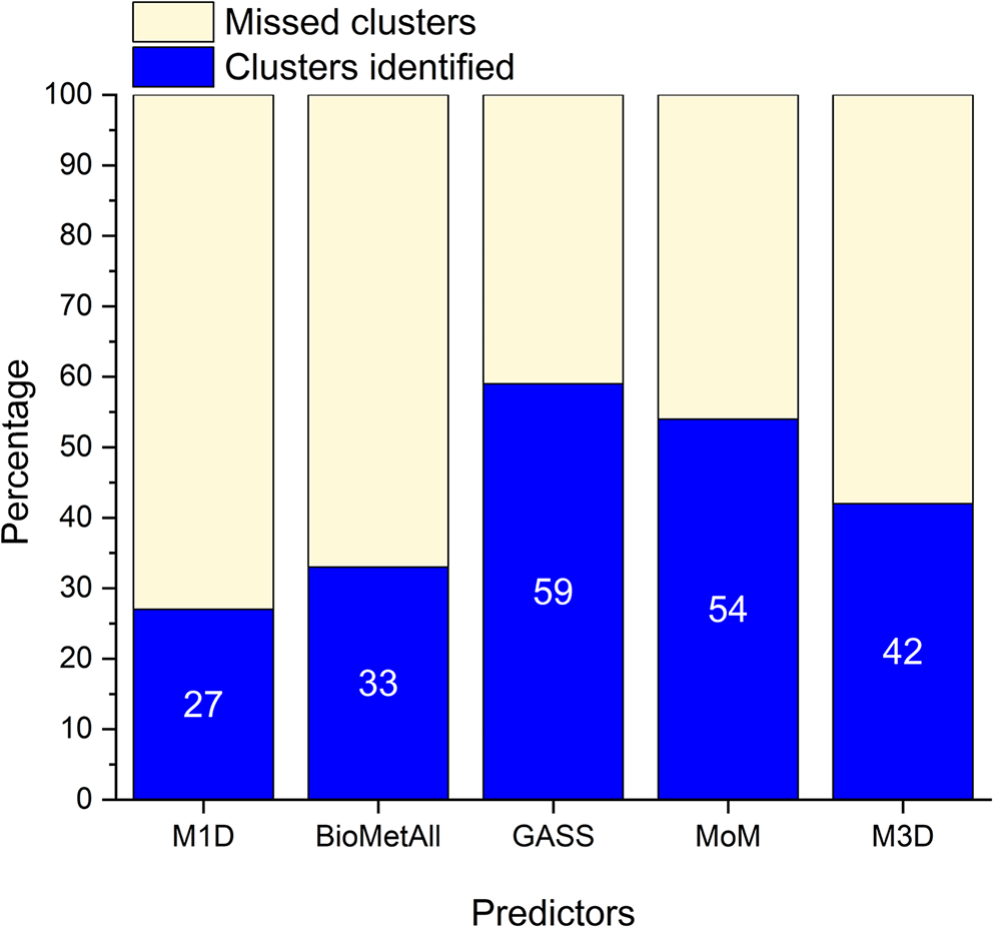
Percentage of CLESs identified by the predictors.

### Impact of Structural Diversity within Families

We selected the top-performing predictors from the previous evaluation, MoM, M3D, and GASS, to investigate their ability to cope with the spread in side chain orientation (Figure 2) within a protein family. By focusing on the CLESs that, based on the violin plots, appear to be particularly affected by this phenomenon, we observed that MoM and GASS are quite tolerant toward structural rearrangements, whereas M3D is impacted by the phenomenon to a larger extent (Figure 5A). Figure 5B,C illustrates how for an apo-site with four Cys residues, these side chains can be spatially close in some structures but point away from each other in other members of the CLES. For this example, the results of GASS and MoM are indeed far better than M3D (Figure 5A, second cluster).

**Figure 5:**
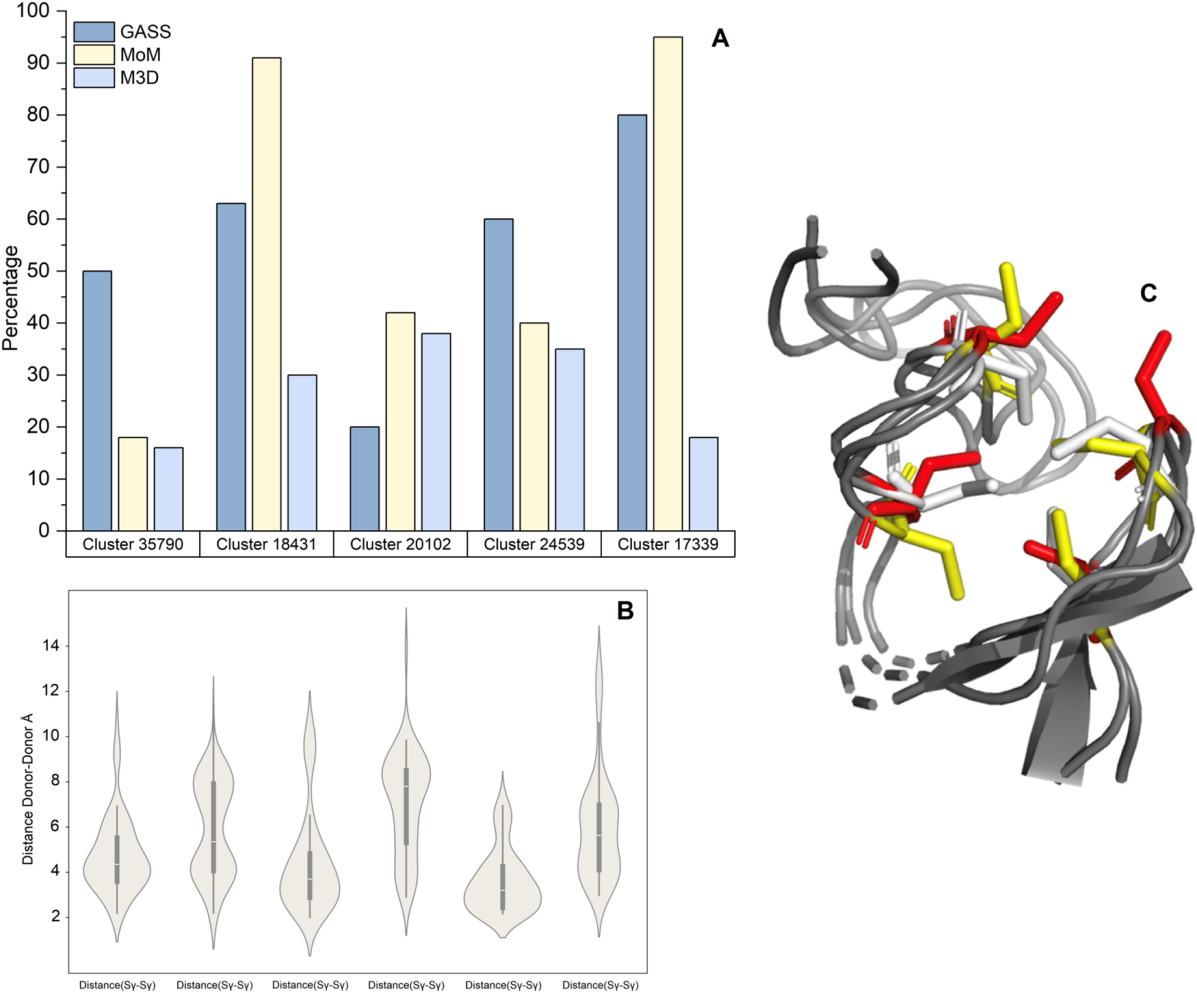
(A) Percentage of correctly predicted sites in CLESs featuring large rearrangements of the MBS. The performance of the three best predictors, each shown in a different color, is computed as the percentage of correctly identified sites over the structures in each CLES. Five different CLESs characterized by a high spread of side chains were selected. (B) Violin plot of CLES 18431. The site of this cluster consists of 4 cysteines, whose donor atoms are the Sγ atoms. (C) View of the 3D structure of the sites in CLES 18431. This panel shows the spread of side chains for three different apo-sites belonging to CLES 18431. Each apo-site is represented by a different color.

### Impact of the Presence of Multiple Metal-Binding Sites and of Redundancy in the Predictions

A specific aspect of metalloproteins is that each structure may contain multiple true MBSs. In fact, our benchmark contained on average 2 sites per structure, going from 1 to 12; 38% of the structures contained a single site (Figure 1). In real life the number of MBS is not known for a novel protein, and thus for an user it would be relevant to understand the behavior of the tools when taking into account a fixed number of predictions for each structure in order to assess whether the predictions of multiple sites in a structure are all equally reliable. We performed this analysis only for the three best predictors (GASS, M3D, MoM). Importantly, if the fixed number of predictions analyzed is lower than the number of real sites in the structure, then the traditional recall (as computed for Figure 3) will always be lower than 100%, introducing a biased view. To provide a fairer evaluation of recall in light of these considerations, we introduced the following formulas

1 $$R\left(\mathrm{top}1\right)=\frac{\mathrm{TP}}{N_{1}^{\mathrm{sites}}+{\frac{1}{2}N}_{2}^{\mathrm{sites}}+{\frac{1}{3}N}_{3}^{\mathrm{sites}}+{\frac{1}{4}N}_{4}^{\mathrm{sites}}+...}$$

2 $$R\left(\mathrm{top}2\right)=\frac{\mathrm{TP}}{N_{1}^{\mathrm{sites}}+N_{2}^{\mathrm{sites}}+{\frac{2}{3}N}_{3}^{\mathrm{sites}}+{\frac{2}{4}N}_{4}^{\mathrm{sites}}+...}$$

3 $$R\left(\mathrm{top}3\right)=\frac{\mathrm{TP}}{N_{1}^{\mathrm{sites}}+N_{2}^{\mathrm{sites}}+N_{3}^{\mathrm{sites}}+{\frac{3}{4}N}_{4}^{\mathrm{sites}}+...}$$

where *N*_1_ is the number of sites in structures with only one site, *N*_2_ is the number of sites in structures with two sites, etc. In practice the denominator of the fractions in eqs 1–3 corresponds to the total number of sites that can be identified given the number of predictions examined.

To define these, we reasoned that if only the prediction with the highest score is considered (Top1), the structures harboring multiple sites would be penalized by the usual Recall equation, because only one site could be predicted. Thus, in Top1 we consider the prediction for a multisite structure successful when any of its sites is correctly identified. With this approach, the number of predictable sites for a two-site structure is effectively halved, whereas for a three-site structure the number of predictable sites is one-third, and so on (eq 1). When computing Top2, all the sites in mono- and two-site structures are predictable, whereas this is true only for two-thirds of the sites in three-site structures (eq 2). This reasoning can be extended to the analysis of any number of output predictions. Note that with monosite structures, it is possible to generate only one correct prediction. An ideal tool thus should output only one prediction with a score better than the threshold. Any additional nonredundant prediction is a FP by definition and thus contributes to decreasing Precision. With the definition of eqs 1–3, the denominator of the fraction increases with increasing number of predictions evaluated. This makes it possible for Recall­(Topm) to decrease upon inclusion of additional predictions in the evaluation, contrary to its commonly observed behavior in binary classification applications. On the other hand, Precision can be computed according to the usual formula, as it corresponds to the percentage of predictions that are correct. For this parameter, predictions that are redundant with respect to a TP can be regarded as TPs as well. The results obtained are shown in Figure 6.

**Figure 6:**
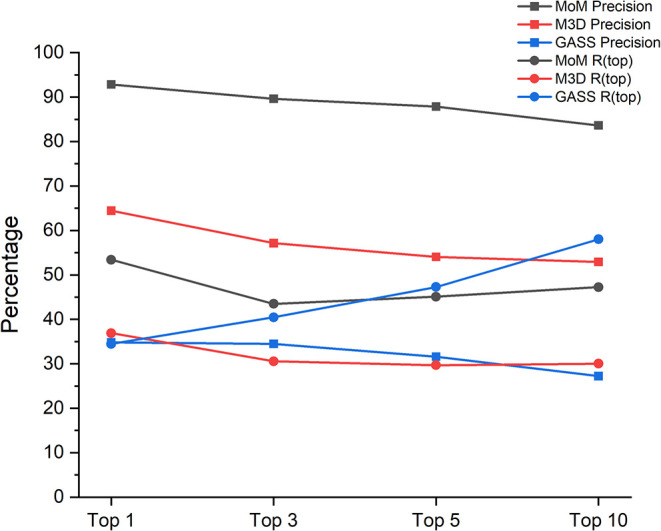
Precision and recall (R­(top)) of the selected predictors as a function of the number of outputs considered.

The Rtop measure ranges between 43 and 53% for MoM, and between 30 and 37% for M3D, with a similar trend as a function of the number of predictions used. The trend is completely different for GASS, whose Rtop increases almost linearly with increasing number of predictions, going from slightly above 34% up to 58% for ten predictions. The *R* top(5) value is similar for MoM and GASS, in agreement with Figure 3. For all predictors, precision decreases by approximately 10% when moving from the top 1 to the top 10 outputs; however MoM had a precision significantly higher than the other programs, with a maximum value of 92% when considering only the first output with respect to about 65% for M3D and 35% for GASS.

In this work, it became apparent that the various predictors could output, albeit to different extents, a number of slightly different predictions for the same site in each structure. To assess the impact of this, we repeated the above analysis by removing all redundant predictions. To do so, for all the correct predictions with *n*-1 ligands in common (where *n* is the number of ligands in the known holo-site), which are redundant TPs, we kept only the best-scoring one. The results are shown in Figure 7.

**Figure 7:**
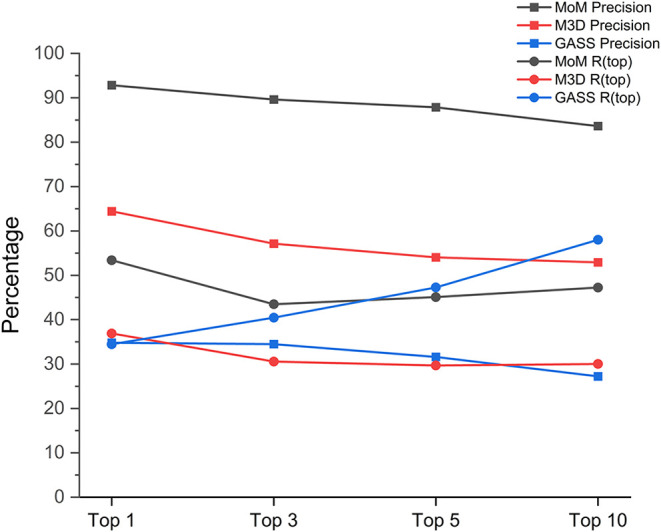
Precision and recall (*R* (top)) of the selected predictors as a function of the number of nonredundant outputs.

This procedure mimics a user who would consider all the predictions involving the same residues but one as identifying the same site. With this approach, the Rtop measure ranges between 45 and 55% for MoM, and between 30 and 40% for M3D, with a similar trend as a function of the number of predictions used. GASS maintains a significantly different trend with Rtop increasing almost linearly from 35% up to almost 60% when including the best 10 predictions in the analysis. In terms of precision, while the situation for the best prediction is unchanged (as there are no redundancies), we can observe a somewhat sharper decline when redundant predictions are excluded for MoM as well as GASS, whereas this change is less pronounced for M3D. In fact, the latter tool is one that produces the lowest number of redundant predictions on average.

The three tools on which we focused featured somewhat different values and trends of precision and recall as a function of the number of predictions included in the analysis. To simplify the overall view of these results, the F-score can be used. Here, we used the F1-score, which is the harmonic mean of precision and recall, to represent the results after removing redundancies (Figure 8).

**Figure 8:**
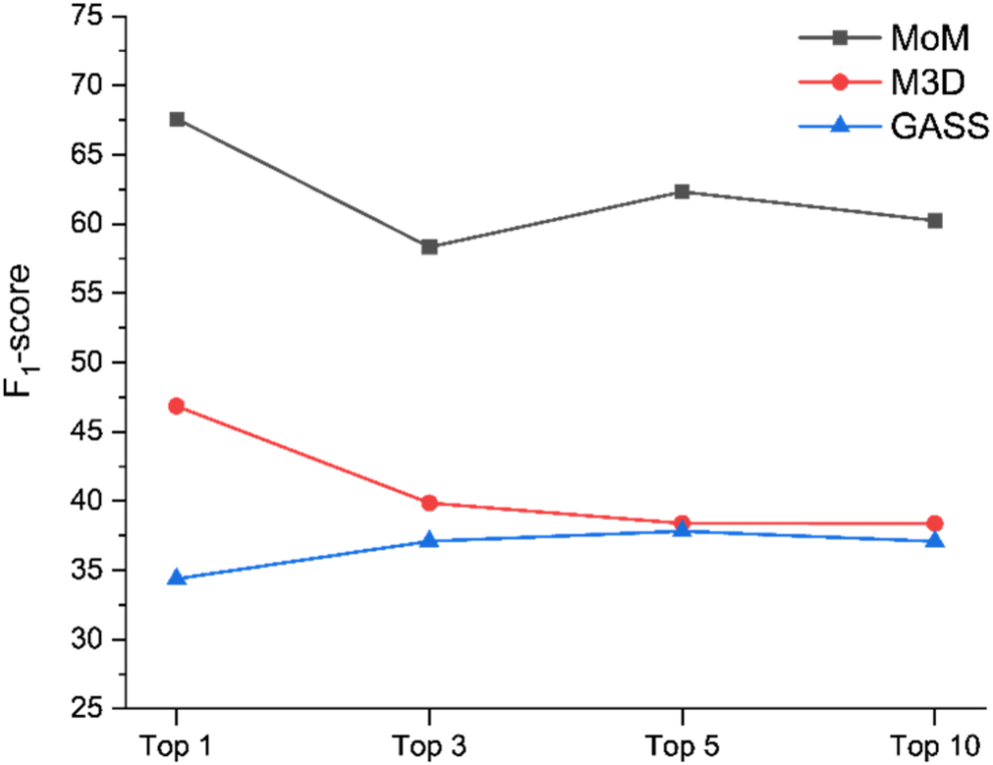
F1-score of the selected predictors as a function of the number of considered outputs. The F1-score has been calculated using the data of Figure 7.

It can be seen that the three programs have similar trends, as the F1-score decreases with increasing number of predictions analyzed. For MoM and M3D, which are the two best-performing tools based on the F1-score, there is a larger drop when extending from 1 to 3 predictions due to the larger number of true sites that ideally should be identified, which adversely affects both their precision and recall. The F1-score of GASS is systematically lower than the other two tools. The F1-score of MoM goes from 68 to 46% with increasing number of predictions, whereas M3D goes from 57 to 38%.

### Predictions for AF Models

In the second benchmark, we selected as input all the proteins from the S. cerevisiae proteome that do not have homologous proteins with a deposited 3D structure in the PDB. For this benchmarking procedure we lack a reference holo structure to evaluate the predictions. Thus, an input protein was considered a metalloprotein if at least two predictors identified the same site. The predictors used in this analysis were MoM, M3D, and GASS, along with AlphaFold 3. For these proteins, model structures were downloaded from the AlphaFold database and filtered on the basis of the average pLDDT over the entire model. A total of 62 sites were identified in the 67 target structures. It is important to note that, unlike the previous benchmark, this analysis also includes true negatives (TN), as some structures were found not to be metalloproteins according to the criterion described above. Of these 62 sites, 27 (44%) were identified by all four predictors, whereas 21 (34%) were predicted by three tools (Figure 9). Eight (13%) sites were identified by only MoM and GASS. The agreement between pairs of tools, excluding AF3, was in the range 69–75%, hence similar for all possible pairs.

**Figure 9:**
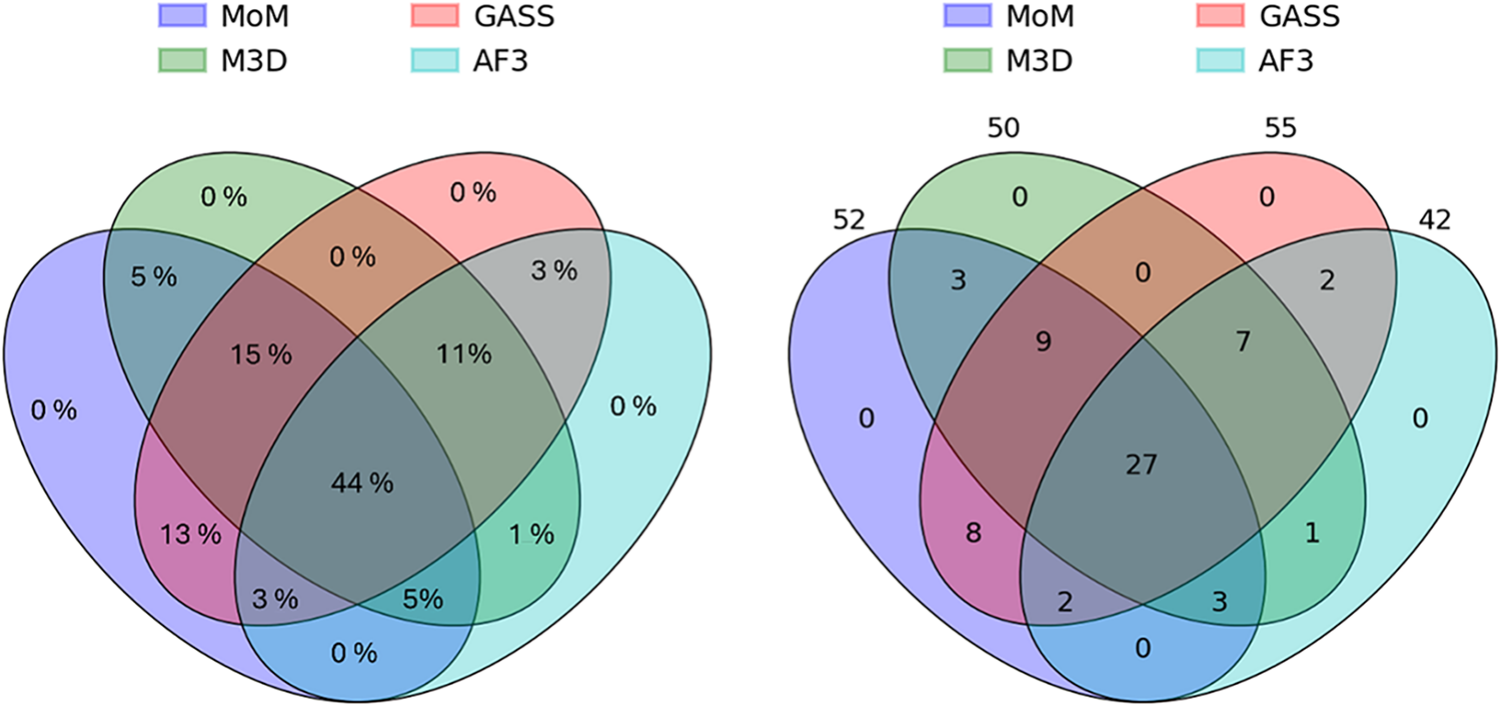
Venn diagram representing the agreement of the predictors for the second benchmark. The diagrams represent the intersections of the true positives (TP) identified by the four tools. The percentages in the left panel were computed with respect to the total of 62 sites identified by at least two predictors.

From the confusion matrix (Table 2) and from Figure 10, we can observe a very satisfactory outcome for all predictors, with M3D and MoM achieving the highest precision (around 90%), whereas GASS and AF3 had similar results (around 70%). GASS, on the other hand, had the highest recall at about 89%, followed by MoM (84%), M3D (81%) and last AF3 at only 68%. As can be seen from the previously shown results, M3D and MoM achieve the best performance in this case as well, with M3D standing out from the others by achieving the highest precision value.

**Table 2:** Confusion Matrix for All Predictors for AF Models

predictor	GASS	MoM	M3D	AF3
TP	55	52	50	42
FP	24	7	4	17
TN	0	12	14	8
FN	0	8	13	12
recall (%)	88.7	83.8	80.6	67.7
precision (%)	69.6	88.1	92.5	71.1

**Figure 10:**
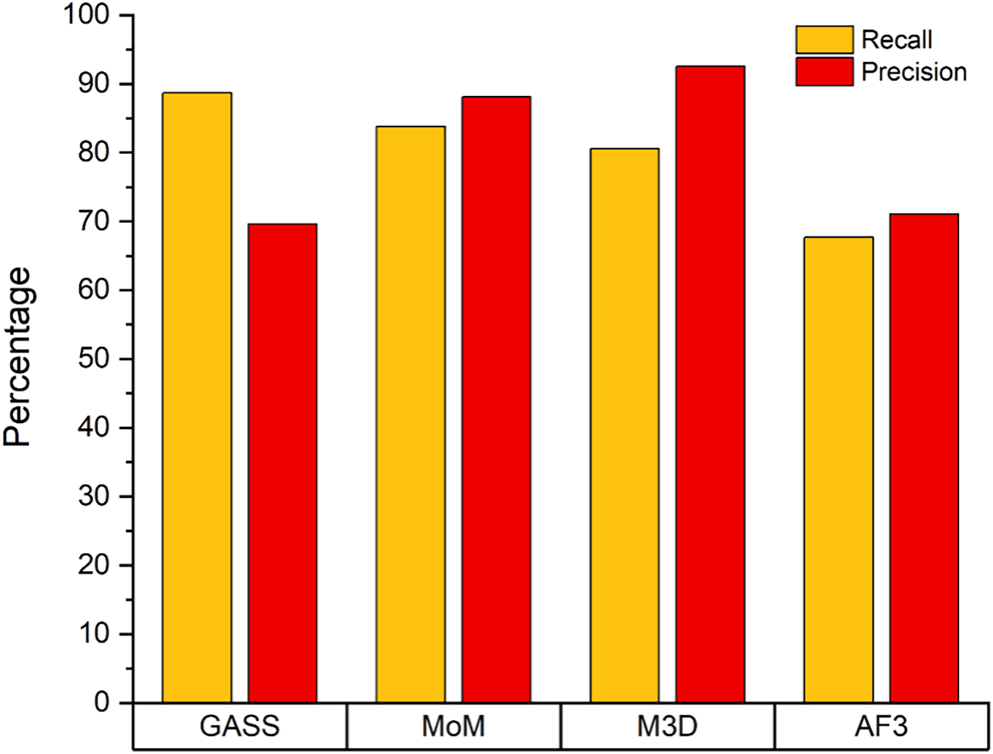
Recall compared with precision for all the predictors. Recall is defined as the ratio between TP and the total number of expected sites in the data set.

## Discussion

As the first step of our work, we benchmarked a number of structure-based predictors of MBS against experimental structures of metalloproteins in their apo form. Structural rearrangements occur upon metalation in apo vs holo proteins, leading to potential differences in the orientation of the side chains at the metal site.^31^ In a cluster (CLES) of apo structures of the same MBS, multiple local energy minima may arise during the crystallization process. This is typically not observed in holo sites, as metal binding imposes order through the geometric constraints of coordination.^33^ The analysis of the CLESs that feature pronounced structural rearrangements revealed that the rearrangement of one residue often is accompanied by movements of other residues within the same binding site. In CLESs with enough sites (typically ≥ 4), this phenomenon becomes quite evident, so that many of the sites exhibiting significant rearrangement can be grouped into subclusters based on the distances between donor atoms. These subclusters represent some of the various relative energy minima that may arise during the crystallization process.

Our results (Figure 3) show that the performance observed here is worse than that reported in the predictors’ respective articles. We can ascribe this to apo structures being a particularly challenging input, owing to their aforementioned structural variations with respect to the holo structure. This observation aligns with the per-CLES performance analysis of Figure 4, which shows that the three best-performing tools can identify only 40 to 60% of the CLESs. The difficulty of this data set is indirectly confirmed by the results obtained in the second part of this study (Figure 9), where all input structures were generated by AlphaFold 2. In this case, the performance was very good and consistent with the results reported in the literature. This is likely because AlphaFold 2 tends to orient the residues in the sites in a configuration relatively close to that of the holo-site,^34^ thus reducing the inherent challenges of apo structures. Notably, in the related task of predicting drug binding modes, AlphaFold models performed comparably to traditional homology models despite their closer similarity to experimental structures.^35^ It is thus possible that zinc­(II) ions constitute a particularly favorable example.

All the predictors are structure-based predictors, but each employs a different criterion for identifying potential MBS within an input structure (see the Methods Section). We observed (Figure 5) that M3D has limitations in accurately identifying sites within all the subclusters of the CLESs that feature a pronounced spread of side chain orientations. MoM and GASS have a better performance under these challenging conditions. The different performance of the predictors can be attributed to the strategy they use for site prediction. MoM and GASS rely on the distance matrix between the α and β carbons of the residues in the potential MBS as their primary predictive parameter. This approach makes them less sensitive to variations in side chain orientation. In contrast, M3D considers the positions of all the atoms within the side chains, which makes it more susceptible to the effects of differing side chain orientations within a potential MBS. It is evident that a large variability between the apo sites within a CLES necessarily implies that certain subsets of distances will deviate significantly from those observed in the metalated form of the site. On the other hand, the global performance of M3D is intermediate between MoM and GASS, indicating that M3D performs very well when the distances and orientations of the residues are close to the holo form.

MoM and M3D tend to generate far fewer predictions than GASS for each input structure, based on the settings specified in the respective articles. Each predictor employs a specific metric to assess the reliability of its outputs; therefore, we investigated whether such a metric can be used meaningfully to rank the results, i.e., as a scoring function. With this approach, we observed that the performance of M3D and MoM is relatively independent of the number of predictions used in the analysis, whereas GASS featured a very strong increase of its recall (*R* (top) values) with increasing number of predictions analyzed. When only the best prediction is taken into account, GASS is outperformed by the M3D and MoM in terms of both recall and precision. When including three predictions in the analysis, the *R* (top) of GASS improves significantly, whereas it drops for the other tools. This can be ascribed to the scoring function of GASS being poorly informative, so that the correct predictions are not necessarily ranked best. This trend becomes more evident for the analysis of up to 10 predictions, when GASS achieves the best *R* (top) of all tools, along with a very poor precision. A combined view of these two parameters is afforded by the F1-score. The latter indicates that providing a limited number of predictions ranked by a reliable scoring function, as done by M3D and MoM, is the best approach. This is indeed also the situation that a user is most likely to appreciate, as it reduces the effort required to interpret the output predictions.

Utilizing computational models of the 3D structure as input is a likely scenario for the use of the predictors discussed here, especially considering AlphaFold’s relatively recent success. We therefore addressed this aspect by investigating a selection of 67 proteins from yeast that have no homologs with an experimental structure deposited in the PDB. As we do not know whether these proteins harbor an MBS, a consensus approach was taken to identify the “true” metalloproteins in this group, leading to an ensemble of 52 proposed ZnPs. In this analysis, AlphaFold 3 was used as well, taking advantage of its ability to predict metal sites, which was not possible with AlphaFold 2. 78% of the sites have been identified by at least three predictors, which appears to be a good consistency. Based on the false positive rates reported in the articles of GASS, M3D and MoM, which are all in the range 10–20%, we would expect that predictions where only two tools are in agreement would have an error rate of less than 5%, whereas the agreement of three tools would correspond to an error rate of about 1%. The pairwise agreement between pairs of predictors was fairly similar, close to or slightly above 70%, suggesting that indeed their radically different designs make the consensus approach meaningful. In practice, based on our second benchmark M3D and MoM have similar performance; the performance of GASS was slightly inferior, especially in terms of precision, whereas that of AF3 was even lower. Notably, M3D and MoM obtained recall and precision values in line with those reported in the respective publications, suggesting that AlphaFold models computed in the absence of the cofactor are a better representation of the holo form of a metalloprotein than the experimental structure of the apo form. Although this is a preliminary observation, due to the small size of the present data set, it is in line with previous observations e.g., for the bulkier iron–sulfur cofactors.^36^ In fact, for the latter systems the effect might be even more dramatic, owing to the large structural changes and increased protein dynamics upon loss of the cofactor.^37,38^

When using AlphaFold models as input, M3D and MoM reach precision values around 90%, which indicates that a computational pipeline that integrates high-quality modeling by AlphaFold with specialized predictors can enable the prediction of entire metalloproteomes. It is also noteworthy that M3D and MoM perform similarly despite the fundamentally different computational approaches they implemented. The use of consensus approaches involving these tools (and others of similar quality) should therefore further increase the reliability of the results. All the tools analyzed in this work are freely downloadable and can be installed with relative ease; a web interface is also available for all of them, except for MoM.

## Supplementary Material



## Notes

List of the apo structures and the corresponding sites used for the benchmark, grouped by CLES number (Table S1); complete list of the selected proteins from the S. cerevisiae proteome that do not have homologous metalloproteins with a deposited 3D structure in the PDB, constituting the second benchmark (Table S2); violin plots of the 15 most populated CLESs (Figures S1–S15) (PDF)
