# Correction to “Benchmarking Zinc-Binding Site Predictors: A Comparative Analysis of Structure-Based Approaches”

**DOI:** 10.1021/acs.jcim.5c01366

**Published:** 2025-07-02

**Authors:** Cosimo Ciofalo, Vincenzo Laveglia, Claudia Andreini, Antonio Rosato

The correct image for Figure 7 is below:

**Figure 7:**
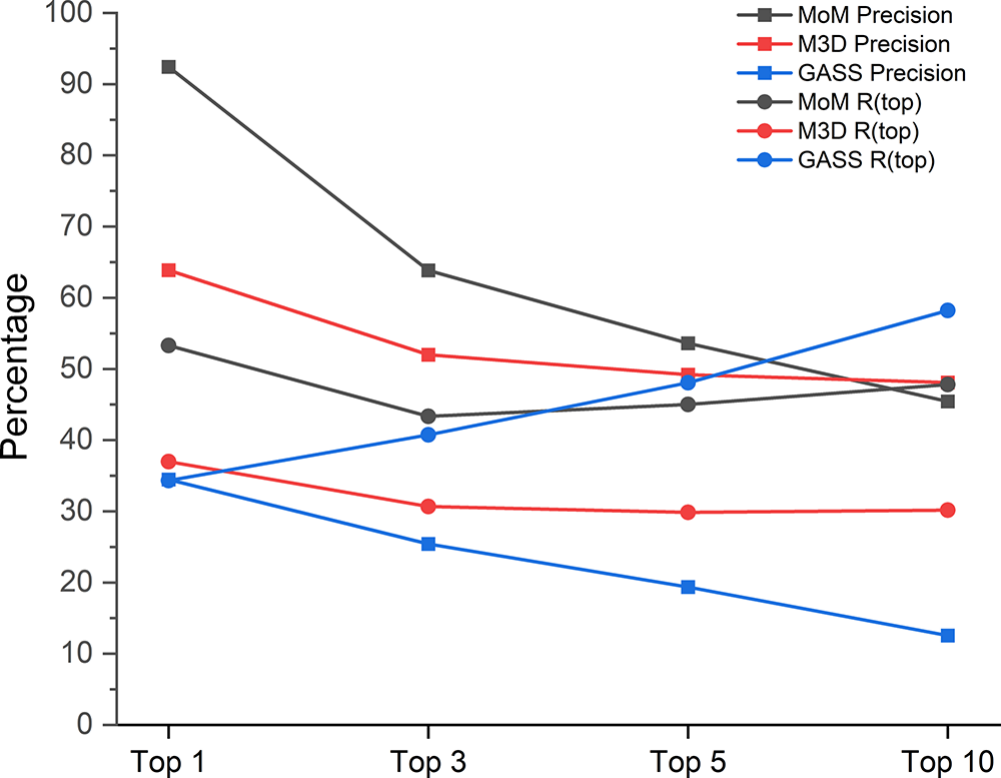
Precision and recall (*R*(top)) of the selected predictors as a function of the number of nonredundant outputs.

