# Supplementary material for: Benchmarking Zinc-Binding Site Predictors: A Comparative Analysis of Structure-Based Approaches
Source: J Chem Inf Model. 2025 May 15;65(10):5205–15. doi: 10.1021/acs.jcim.5c00549 (PMC12117554; doi:10.1021/acs.jcim.5c00549)
Supplement: Supplementary file 1 [file ci5c00549_si_001.pdf]

Benchmarking Zinc-Binding Site Predictors: A Comparative Analysis of Structure-Based Approaches

Cosimo Ciofalo[1,2], Vincenzo Laveglia[1], Claudia Andreini[1,2,3,*], Antonio Rosato[1,2,3,*]

[1] Department of Chemistry, University of Florence, Via della Lastruccia 3, 50019 Sesto Fiorentino, Italy.

[2] Magnetic Resonance Center (CERM), University of Florence, Via Luigi Sacconi 6, 50019 Sesto Fiorentino, Italy

[3] Consorzio Interuniversitario di Risonanze Magnetiche di Metallo Proteine, Via Luigi Sacconi 6, 50019 Sesto Fiorentino, Italy.

Supporting Information

Table S1. List of the apo structures and the corresponding sites used for the benchmark, grouped by CLES number

CLES	Apo_id	Metal site
16102	1lmz	CYS:4:A HIS:17:A HIS:175:A CYS:179:A
16144	1v7o	HIS:9:B HIS:13:B CYS:116:B
16144	3rhu	HIS:9:A HIS:13:A CYS:116:A
16144	3rhu	HIS:9:B HIS:13:B CYS:116:B
16144	1v7o	HIS:9:A HIS:13:A CYS:116:A
16236	4kzz	CYS:121:f CYS:126:f CYS:141:f CYS:144:f
16236	4d61	CYS:121:f CYS:126:f CYS:141:f CYS:144:f
16236	4kzy	CYS:121:f CYS:126:f CYS:141:f CYS:144:f
16236	4kzx	CYS:121:f CYS:126:f CYS:141:f CYS:144:f
16236	4d5l	CYS:121:f CYS:126:f CYS:141:f CYS:144:f
16466	5hx4	HIS:249:A CYS:280:A CYS:283:A
16492	1tzp	HIS:110:A HIS:113:A ASP:120:A HIS:211:A
16492	1tzp	HIS:110:B HIS:113:B ASP:120:B HIS:211:B
16612	5kbp	HIS:13:B ASP:15:B ASP:125:B HIS:350:B
16612	5kbp	HIS:13:A ASP:15:A ASP:125:A HIS:350:A
16944	3j38	CYS:37:b CYS:56:b CYS:59:b
16944	4kzy	CYS:37:b CYS:56:b CYS:59:b
16944	4kzx	CYS:37:b CYS:56:b CYS:59:b
16944	4d61	CYS:37:b CYS:56:b CYS:59:b
16944	3j3a	CYS:37:b CYS:56:b CYS:59:b
16944	4kzz	CYS:37:b CYS:56:b CYS:59:b
16944	4d5l	CYS:37:b CYS:56:b CYS:59:b

16944	5k0y	CYS:37:Y CYS:56:Y CYS:59:Y
16944	3izb	CYS:37:X CYS:56:X CYS:59:X
16944	3zey	CYS:38:W CYS:57:W CYS:60:W
16944	3iz6	CYS:39:X CYS:58:X CYS:61:X
16958	5u0s	CYS:75:i CYS:78:i CYS:103:i CYS:106:i
17016	1nij	GLU:37:A GLU:42:A CYS:66:A
17142	3gfb	CYS:97:B CYS:100:B CYS:103:B CYS:111:B
17142	3gfb	CYS:97:A CYS:100:A CYS:103:A CYS:111:A
17142	3gfb	CYS:97:C CYS:100:C CYS:103:C CYS:111:C
17142	3gfb	CYS:97:D CYS:100:D CYS:103:D CYS:111:D
17311	4jqs	HIS:48:B HIS:120:B GLU:178:B HIS:234:B
17311	4jqs	HIS:48:A HIS:120:A GLU:178:A HIS:234:A
17311	4jqs	HIS:48:C HIS:120:C GLU:178:C HIS:234:C
17321	4gd0	CYS:318:A CYS:320:A CYS:323:A HIS:349:A
17339	2ipk	ASP:83:D HIS:118:D HIS:122:D
17339	1sjh	ASP:83:D HIS:118:D HIS:122:D
17339	2aq1	ASP:83:B HIS:116:B HIS:120:B
17339	2aq3	ASP:83:B HIS:116:B HIS:120:B
17339	3byt	ASP:83:H HIS:116:H HIS:120:H
17339	2aq1	ASP:83:H HIS:116:H HIS:120:H
17339	1jwu	ASP:83:D HIS:118:D HIS:122:D
17339	3byt	ASP:83:D HIS:116:D HIS:120:D
17339	1jwm	ASP:83:D HIS:118:D HIS:122:D
17339	2aq1	ASP:83:F HIS:116:F HIS:120:F
17339	1jck	ASP:83:D HIS:118:D HIS:122:D

1733 9	1klu	ASP:83:D HIS:118:D HIS:122:D
1733 9	2aq1	ASP:83:D HIS:116:D HIS:120:D
1733 9	3bzd	ASP:83:B HIS:116:B HIS:120:B
1733 9	3byt	ASP:83:F HIS:116:F HIS:120:F
1733 9	2aq3	ASP:83:F HIS:116:F HIS:120:F
1733 9	1se2	ASP:83:A HIS:118:A HIS:122:A
1733 9	1jck	ASP:83:B HIS:118:B HIS:122:B
1733 9	1jws	ASP:83:D HIS:118:D HIS:122:D
1733 9	1pyw	ASP:83:D HIS:118:D HIS:122:D
1733 9	2aq3	ASP:83:D HIS:116:D HIS:120:D
1733 9	1sje	ASP:83:D HIS:118:D HIS:122:D
1733 9	1t5x	ASP:83:D HIS:118:D HIS:122:D
1733 9	2aq3	ASP:83:H HIS:116:H HIS:120:H
1733 9	3byy	ASP:83:B HIS:116:B HIS:120:B
1733 9	3byt	ASP:83:B HIS:116:B HIS:120:B
1733 9	1klg	ASP:83:D HIS:118:D HIS:122:D
1741 0	3t6j	HIS:450:A HIS:455:A GLU:508:A
1741 0	3t6b	HIS:450:B HIS:455:B GLU:508:B
1741 0	3t6b	HIS:450:A HIS:455:A GLU:508:A
1741 5	3ppf	HIS:657:A CYS:659:A CYS:739:A
1741 5	3l7r	HIS:630:A CYS:632:A CYS:715:A
1741 5	1t7l	HIS:618:B CYS:620:B CYS:704:B
1741 5	1xr2	HIS:618:B CYS:620:B CYS:704:B
1741 5	1t7l	HIS:618:A CYS:620:A CYS:704:A
1741 5	1xr2	HIS:618:A CYS:620:A CYS:704:A

17415	3pph	HIS:657:A CYS:659:A CYS:739:A
17415	3pph	HIS:657:B CYS:659:B CYS:739:B
17415	2nq5	HIS:630:A CYS:632:A CYS:715:A
17508	1ro4	CYS:15:A HIS:18:A CYS:34:A CYS:37:A
17552	5c44	CYS:31:L GLU:33:L CYS:34:L CYS:48:L CYS:51:L
17552	3j1n	CYS:31:L GLU:33:L CYS:34:L CYS:48:L CYS:51:L
17552	5u0s	CYS:24:I CYS:27:I CYS:41:I CYS:44:I
17567	5esz	ASP:120:C HIS:122:C HIS:207:C
17567	3re3	ASP:10:A HIS:12:A HIS:45:A
17567	5esz	ASP:120:G HIS:122:G HIS:207:G
17567	3re3	ASP:10:B HIS:12:B HIS:45:B
17567	3re3	ASP:10:D HIS:12:D HIS:45:D
17567	3re3	ASP:10:C HIS:12:C HIS:45:C
17610	1r3b	CYS:78:A CYS:83:A HIS:160:A HIS:165:A
17751	4jid	HIS:160:B ASP:167:B GLU:216:B HIS:219:B
17751	4jid	HIS:160:A ASP:167:A GLU:216:A HIS:219:A
17910	3q7g	HIS:376:A HIS:426:A HIS:430:A
17910	3pmr	HIS:438:B HIS:488:B HIS:492:B
17910	4rd9	HIS:376:A HIS:426:A HIS:430:A
17910	3q7l	HIS:376:A HIS:426:A HIS:430:A
17910	3q7l	HIS:376:B HIS:426:B HIS:430:B
17910	3pmr	HIS:438:A HIS:488:A HIS:492:A
17910	3nyj	HIS:438:A HIS:488:A HIS:492:A
17910	3q7g	HIS:376:B HIS:426:B HIS:430:B
17910	3qmk	HIS:376:A HIS:426:A HIS:430:A

1791<br>0	4rda	HIS:376:A HIS:426:A HIS:430:A
1791<br>0	4rda	HIS:376:B HIS:426:B HIS:430:B
1791<br>0	3qmk	HIS:376:B HIS:426:B HIS:430:B
1791<br>0	4rd9	HIS:376:B HIS:426:B HIS:430:B
1791<br>0	3nyl	HIS:438:A HIS:488:A HIS:492:A
1792<br>3	1rw0	HIS:71:A CYS:107:A HIS:124:A
1792<br>3	1rw0	HIS:71:B CYS:107:B HIS:124:B
1792<br>3	1rv9	HIS:82:A CYS:118:A HIS:135:A
1792<br>3	1z9t	HIS:71:A CYS:107:A HIS:124:A
1803<br>7	2y3u	HIS:523:A HIS:527:A GLU:555:A
1823<br>5	3glh	CYS:64:G CYS:73:G CYS:76:G CYS:79:G
1823<br>5	3glh	CYS:50:E CYS:59:E CYS:62:E CYS:65:E
1823<br>5	3glh	CYS:64:M CYS:73:M CYS:76:M CYS:79:M
1823<br>5	3glh	CYS:50:O CYS:59:O CYS:62:O CYS:65:O
1823<br>5	3glh	CYS:64:N CYS:73:N CYS:76:N CYS:79:N
1823<br>5	3glh	CYS:64:I CYS:73:I CYS:76:I CYS:79:I
1823<br>5	3glh	CYS:64:D CYS:73:D CYS:76:D CYS:79:D
1823<br>5	3glh	CYS:50:J CYS:59:J CYS:62:J CYS:65:J
1823<br>5	3glh	CYS:64:C CYS:73:C CYS:76:C CYS:79:C
1823<br>5	3glh	CYS:64:L CYS:73:L CYS:76:L CYS:79:L
1823<br>5	3glh	CYS:64:B CYS:73:B CYS:76:B CYS:79:B
1823<br>5	3glh	CYS:64:H CYS:73:H CYS:76:H CYS:79:H
1831<br>9	5e6j	CYS:190:A CYS:193:A CYS:225:A CYS:227:A
1843<br>1	2y13	CYS:33:5 CYS:36:5 CYS:46:5 CYS:49:5
1843<br>1	2b66	CYS:33:5 CYS:36:5 CYS:46:5 CYS:49:5

18431	4jux	CYS:33:5 CYS:36:5 CYS:46:5 CYS:49:5
18431	1yl3	CYS:33:5 CYS:36:5 CYS:46:5 CYS:49:5
18431	1vor	CYS:33:2 CYS:36:2 CYS:46:2 CYS:49:2
18431	2y17	CYS:33:5 CYS:36:5 CYS:46:5 CYS:49:5
18431	1vsa	CYS:33:Y CYS:36:Y CYS:46:Y CYS:49:Y
18431	1voy	CYS:33:2 CYS:36:2 CYS:46:2 CYS:49:2
18431	2j03	CYS:33:5 CYS:36:5 CYS:46:5 CYS:49:5
18431	3i8i	CYS:33:5 CYS:36:5 CYS:46:5 CYS:49:5
18431	2wrl	CYS:33:5 CYS:36:5 CYS:46:5 CYS:49:5
18431	4k0m	CYS:33:5 CYS:36:5 CYS:46:5 CYS:49:5
18431	4kdb	CYS:33:5 CYS:36:5 CYS:46:5 CYS:49:5
18431	4kd2	CYS:33:5 CYS:36:5 CYS:46:5 CYS:49:5
18431	4ejc	CYS:33:5 CYS:36:5 CYS:46:5 CYS:49:5
18431	3uz8	CYS:33:5 CYS:36:5 CYS:46:5 CYS:49:5
18431	1vp0	CYS:33:2 CYS:36:2 CYS:46:2 CYS:49:2
18431	2hgu	CYS:33:4 CYS:36:4 CYS:46:4 CYS:49:4
18431	3uz1	CYS:33:5 CYS:36:5 CYS:46:5 CYS:49:5
18431	2wdi	CYS:33:5 CYS:36:5 CYS:46:5 CYS:49:5
18431	2y0v	CYS:33:5 CYS:36:5 CYS:46:5 CYS:49:5
18431	2xg2	CYS:33:5 CYS:36:5 CYS:46:5 CYS:49:5
18431	3tvh	CYS:33:5 CYS:36:5 CYS:46:5 CYS:49:5
18431	2y0x	CYS:33:5 CYS:36:5 CYS:46:5 CYS:49:5
18431	3i9c	CYS:33:5 CYS:36:5 CYS:46:5 CYS:49:5
18431	3fin	CYS:33:5 CYS:36:5 CYS:46:5 CYS:49:5
18431	2wh2	CYS:33:5 CYS:36:5 CYS:46:5 CYS:49:5

1843 1	4b8i	CYS:33:5 CYS:36:5 CYS:46:5 CYS:49:5
1843 1	2v49	CYS:33:5 CYS:36:5 CYS:46:5 CYS:49:5
1843 1	4g5w	CYS:33:5 CYS:36:5 CYS:46:5 CYS:49:5
1843 1	3zn9	CYS:33:5 CYS:36:5 CYS:46:5 CYS:49:5
1843 1	3zne	CYS:33:5 CYS:36:5 CYS:46:5 CYS:49:5
1843 1	2xux	CYS:33:5 CYS:36:5 CYS:46:5 CYS:49:5
1843 1	2zjr	CYS:33:Z CYS:36:Z CYS:46:Z CYS:49:Z
1843 1	4k0q	CYS:33:5 CYS:36:5 CYS:46:5 CYS:49:5
1843 1	4g5u	CYS:33:5 CYS:36:5 CYS:46:5 CYS:49:5
1843 1	2x9u	CYS:33:5 CYS:36:5 CYS:46:5 CYS:49:5
1843 1	3v6x	CYS:33:5 CYS:36:5 CYS:46:5 CYS:49:5
1843 1	3v6w	CYS:33:5 CYS:36:5 CYS:46:5 CYS:49:5
1843 1	2wrj	CYS:33:5 CYS:36:5 CYS:46:5 CYS:49:5
1843 1	2y11	CYS:33:5 CYS:36:5 CYS:46:5 CYS:49:5
1843 1	2v47	CYS:33:5 CYS:36:5 CYS:46:5 CYS:49:5
1843 1	2zjq	CYS:33:Z CYS:36:Z CYS:46:Z CYS:49:Z
1843 1	4kbu	CYS:33:5 CYS:36:5 CYS:46:5 CYS:49:5
1843 1	2j01	CYS:33:5 CYS:36:5 CYS:46:5 CYS:49:5
1843 1	4kcz	CYS:33:5 CYS:36:5 CYS:46:5 CYS:49:5
1843 1	4ejb	CYS:33:5 CYS:36:5 CYS:46:5 CYS:49:5
1843 1	3i9e	CYS:33:5 CYS:36:5 CYS:46:5 CYS:49:5
1843 1	4kd9	CYS:33:5 CYS:36:5 CYS:46:5 CYS:49:5
1843 1	1vou	CYS:33:2 CYS:36:2 CYS:46:2 CYS:49:2
1843 1	3uz2	CYS:33:5 CYS:36:5 CYS:46:5 CYS:49:5
1843 1	2wro	CYS:33:5 CYS:36:5 CYS:46:5 CYS:49:5

1843<br>1	2xqe	CYS:33:5 CYS:36:5 CYS:46:5 CYS:49:5
1843<br>1	4kdh	CYS:33:5 CYS:36:5 CYS:46:5 CYS:49:5
1843<br>1	2wdn	CYS:33:5 CYS:36:5 CYS:46:5 CYS:49:5
1843<br>1	4ioc	CYS:33:Z CYS:36:Z CYS:46:Z CYS:49:Z
1843<br>1	4b8g	CYS:33:5 CYS:36:5 CYS:46:5 CYS:49:5
1843<br>1	2xtg	CYS:33:5 CYS:36:5 CYS:46:5 CYS:49:5
1843<br>1	2y19	CYS:33:5 CYS:36:5 CYS:46:5 CYS:49:5
1843<br>1	4kbw	CYS:33:5 CYS:36:5 CYS:46:5 CYS:49:5
1843<br>1	2b9n	CYS:33:5 CYS:36:5 CYS:46:5 CYS:49:5
1843<br>1	2y0z	CYS:33:5 CYS:36:5 CYS:46:5 CYS:49:5
1843<br>1	3uxq	CYS:33:5 CYS:36:5 CYS:46:5 CYS:49:5
1843<br>1	3uzn	CYS:33:5 CYS:36:5 CYS:46:5 CYS:49:5
1843<br>1	3tve	CYS:33:5 CYS:36:5 CYS:46:5 CYS:49:5
1843<br>1	2wdj	CYS:33:5 CYS:36:5 CYS:46:5 CYS:49:5
1843<br>1	3zvp	CYS:33:5 CYS:36:5 CYS:46:5 CYS:49:5
1843<br>1	4abs	CYS:33:5 CYS:36:5 CYS:46:5 CYS:49:5
1843<br>1	4kdk	CYS:33:5 CYS:36:5 CYS:46:5 CYS:49:5
1843<br>1	2wdl	CYS:33:5 CYS:36:5 CYS:46:5 CYS:49:5
1843<br>1	3uzk	CYS:33:5 CYS:36:5 CYS:46:5 CYS:49:5
1843<br>1	2y15	CYS:33:5 CYS:36:5 CYS:46:5 CYS:49:5
1843<br>1	3uye	CYS:33:5 CYS:36:5 CYS:46:5 CYS:49:5
1843<br>1	4g5n	CYS:33:5 CYS:36:5 CYS:46:5 CYS:49:5
1843<br>1	1vow	CYS:33:2 CYS:36:2 CYS:46:2 CYS:49:2
1843<br>1	2b9p	CYS:33:5 CYS:36:5 CYS:46:5 CYS:49:5
1843<br>1	3uzh	CYS:33:5 CYS:36:5 CYS:46:5 CYS:49:5

1843<br>1	3i8f	CYS:33:5 CYS:36:5 CYS:46:5 CYS:49:5
1843<br>1	4ioa	CYS:33:Z CYS:36:Z CYS:46:Z CYS:49:Z
1843<br>1	2x9s	CYS:33:5 CYS:36:5 CYS:46:5 CYS:49:5
1843<br>1	4g5l	CYS:33:5 CYS:36:5 CYS:46:5 CYS:49:5
1843<br>1	1vsp	CYS:33:Y CYS:36:Y CYS:46:Y CYS:49:Y
1843<br>1	3uyg	CYS:33:5 CYS:36:5 CYS:46:5 CYS:49:5
1843<br>1	2hgj	CYS:33:4 CYS:36:4 CYS:46:4 CYS:49:4
1843<br>1	2wrr	CYS:33:5 CYS:36:5 CYS:46:5 CYS:49:5
1843<br>1	2wh4	CYS:33:5 CYS:36:5 CYS:46:5 CYS:49:5
1843<br>1	4io9	CYS:33:Z CYS:36:Z CYS:46:Z CYS:49:Z
1843<br>1	3uxr	CYS:33:5 CYS:36:5 CYS:46:5 CYS:49:5
1843<br>1	2xg0	CYS:33:5 CYS:36:5 CYS:46:5 CYS:49:5
1843<br>1	3cf5	CYS:33:Y CYS:36:Y CYS:46:Y CYS:49:Y
1843<br>1	3uzf	CYS:33:5 CYS:36:5 CYS:46:5 CYS:49:5
1843<br>1	3uz9	CYS:33:5 CYS:36:5 CYS:46:5 CYS:49:5
1843<br>1	2hgq	CYS:33:4 CYS:36:4 CYS:46:4 CYS:49:4
1864<br>8	2js4	CYS:11:A CYS:14:A CYS:29:A ASP:32:A
1880<br>0	4li7	CYS:1234:B HIS:1237:B CYS:1242:B<br>CYS:1245:B
1880<br>0	4li7	CYS:1234:A HIS:1237:A CYS:1242:A<br>CYS:1245:A
1920<br>4	4wto	CYS:109:D CYS:114:D CYS:138:D CYS:141:D
1920<br>4	4wto	CYS:109:B CYS:114:B CYS:138:B CYS:141:B
1940<br>7	2nxu	CYS:107:A CYS:110:A CYS:128:A CYS:131:A
1940<br>7	5jbh	CYS:106:8 CYS:109:8 CYS:127:8 CYS:130:8
1940<br>7	3cw2	CYS:106:M CYS:109:M CYS:127:M<br>CYS:130:M
1974<br>2	2g47	HIS:108:B HIS:112:B GLU:189:B

1974 2	3tuv	HIS:108:A HIS:112:A GLU:189:A
1974 2	2g48	HIS:108:A HIS:112:A GLU:189:A
1974 2	2g49	HIS:108:B HIS:112:B GLU:189:B
1974 2	3p7o	HIS:108:A HIS:112:A GLU:189:A
1974 2	2jbu	HIS:108:B HIS:112:B GLU:189:B
1974 2	2g56	HIS:108:A HIS:112:A GLU:189:A
1974 2	3ami	HIS:77:A HIS:81:A GLU:157:A
1974 2	2g56	HIS:108:B HIS:112:B GLU:189:B
1974 2	2g49	HIS:108:A HIS:112:A GLU:189:A
1974 2	2jbu	HIS:108:A HIS:112:A GLU:189:A
1974 2	2g47	HIS:108:A HIS:112:A GLU:189:A
1974 2	3ami	HIS:77:B HIS:81:B GLU:157:B
1974 2	2g48	HIS:108:B HIS:112:B GLU:189:B
2010 2	4jux	CYS:42:4 CYS:44:4 CYS:62:4 CYS:65:4
2010 2	2wdj	CYS:42:4 CYS:44:4 CYS:62:4 CYS:65:4
2010 2	3j0w	CYS:16:2 CYS:18:2 CYS:37:2 CYS:40:2
2010 2	3i9e	CYS:16:4 CYS:18:4 CYS:36:4 CYS:39:4
2010 2	2hgu	CYS:16:3 CYS:18:3 CYS:36:3 CYS:39:3
2010 2	3izt	CYS:16:b CYS:18:b CYS:37:b CYS:40:b
2010 2	2y13	CYS:16:4 CYS:18:4 CYS:36:4 CYS:39:4
2010 2	2y19	CYS:16:4 CYS:18:4 CYS:36:4 CYS:39:4
2010 2	4ejb	CYS:42:4 CYS:44:4 CYS:62:4 CYS:65:4
2010 2	3v6w	CYS:42:4 CYS:44:4 CYS:62:4 CYS:65:4
2010 2	2y0v	CYS:16:4 CYS:18:4 CYS:36:4 CYS:39:4
2010 2	2j03	CYS:16:4 CYS:18:4 CYS:36:4 CYS:39:4

2010 2	2j01	CYS:16:4 CYS:18:4 CYS:36:4 CYS:39:4
2010 2	3uz2	CYS:16:4 CYS:18:4 CYS:36:4 CYS:39:4
2010 2	3i8f	CYS:16:4 CYS:18:4 CYS:36:4 CYS:39:4
2010 2	3uz1	CYS:16:4 CYS:18:4 CYS:36:4 CYS:39:4
2010 2	2y0x	CYS:16:4 CYS:18:4 CYS:36:4 CYS:39:4
2010 2	3tvh	CYS:16:4 CYS:18:4 CYS:36:4 CYS:39:4
2010 2	2wdl	CYS:42:4 CYS:44:4 CYS:62:4 CYS:65:4
2010 2	3uzf	CYS:16:4 CYS:18:4 CYS:36:4 CYS:39:4
2010 2	3j0t	CYS:16:2 CYS:18:2 CYS:37:2 CYS:40:2
2010 2	4g5w	CYS:16:4 CYS:18:4 CYS:36:4 CYS:39:4
2010 2	3izu	CYS:16:b CYS:18:b CYS:37:b CYS:40:b
2010 2	2wrr	CYS:16:4 CYS:18:4 CYS:36:4 CYS:39:4
2010 2	3tve	CYS:16:4 CYS:18:4 CYS:36:4 CYS:39:4
2010 2	3uyg	CYS:16:4 CYS:18:4 CYS:36:4 CYS:39:4
2010 2	2y11	CYS:16:4 CYS:18:4 CYS:36:4 CYS:39:4
2010 2	3uye	CYS:16:4 CYS:18:4 CYS:36:4 CYS:39:4
2010 2	3uxq	CYS:16:4 CYS:18:4 CYS:36:4 CYS:39:4
2010 2	2x9u	CYS:16:4 CYS:18:4 CYS:36:4 CYS:39:4
2010 2	3uzk	CYS:16:4 CYS:18:4 CYS:36:4 CYS:39:4
2010 2	3j37	CYS:16:4 CYS:18:4 CYS:37:4 CYS:40:4
2010 2	2v49	CYS:16:4 CYS:18:4 CYS:36:4 CYS:39:4
2010 2	2v47	CYS:16:4 CYS:18:4 CYS:36:4 CYS:39:4
2010 2	2wrj	CYS:16:4 CYS:18:4 CYS:36:4 CYS:39:4
2010 2	4b8i	CYS:16:4 CYS:18:4 CYS:36:4 CYS:39:4
2010 2	2wdn	CYS:42:4 CYS:44:4 CYS:62:4 CYS:65:4

2010 2	3j11	CYS:16:2 CYS:18:2 CYS:37:2 CYS:40:2
2010 2	2xg2	CYS:16:4 CYS:18:4 CYS:36:4 CYS:39:4
2010 2	2xqe	CYS:16:4 CYS:18:4 CYS:36:4 CYS:39:4
2010 2	3uz8	CYS:16:4 CYS:18:4 CYS:36:4 CYS:39:4
2010 2	4abs	CYS:16:4 CYS:18:4 CYS:36:4 CYS:39:4
2010 2	2wdi	CYS:42:4 CYS:44:4 CYS:62:4 CYS:65:4
2010 2	3j12	CYS:16:2 CYS:18:2 CYS:37:2 CYS:40:2
2010 2	3uzn	CYS:16:4 CYS:18:4 CYS:36:4 CYS:39:4
2010 2	4b8g	CYS:16:4 CYS:18:4 CYS:36:4 CYS:39:4
2010 2	4g5u	CYS:16:4 CYS:18:4 CYS:36:4 CYS:39:4
2010 2	2xtg	CYS:16:4 CYS:18:4 CYS:36:4 CYS:39:4
2010 2	2wrl	CYS:16:4 CYS:18:4 CYS:36:4 CYS:39:4
2010 2	2y17	CYS:16:4 CYS:18:4 CYS:36:4 CYS:39:4
2010 2	3uxr	CYS:16:4 CYS:18:4 CYS:36:4 CYS:39:4
2010 2	2wro	CYS:16:4 CYS:18:4 CYS:36:4 CYS:39:4
2010 2	4ejc	CYS:16:4 CYS:18:4 CYS:36:4 CYS:39:4
2010 2	3j14	CYS:16:2 CYS:18:2 CYS:37:2 CYS:40:2
2010 2	2xg0	CYS:16:4 CYS:18:4 CYS:36:4 CYS:39:4
2010 2	3zne	CYS:16:4 CYS:18:4 CYS:36:4 CYS:39:4
2010 2	2y0z	CYS:16:4 CYS:18:4 CYS:36:4 CYS:39:4
2010 2	3i8i	CYS:16:4 CYS:18:4 CYS:36:4 CYS:39:4
2010 2	4g5n	CYS:16:4 CYS:18:4 CYS:36:4 CYS:39:4
2010 2	3uz9	CYS:16:4 CYS:18:4 CYS:36:4 CYS:39:4
2010 2	3zvp	CYS:16:4 CYS:18:4 CYS:36:4 CYS:39:4
2010 2	2wh2	CYS:42:4 CYS:44:4 CYS:62:4 CYS:65:4

2010<br>2	2hgj	CYS:16:3 CYS:18:3 CYS:36:3 CYS:39:3
2010<br>2	3zn9	CYS:16:4 CYS:18:4 CYS:36:4 CYS:39:4
2010<br>2	3v6x	CYS:42:4 CYS:44:4 CYS:62:4 CYS:65:4
2010<br>2	4g5l	CYS:16:4 CYS:18:4 CYS:36:4 CYS:39:4
2010<br>2	3uzh	CYS:16:4 CYS:18:4 CYS:36:4 CYS:39:4
2010<br>2	3e1d	CYS:16:S CYS:18:S CYS:37:S CYS:40:S
2010<br>2	3e1b	CYS:16:S CYS:18:S CYS:37:S CYS:40:S
2010<br>2	2x9s	CYS:16:4 CYS:18:4 CYS:36:4 CYS:39:4
2010<br>2	3i9c	CYS:16:4 CYS:18:4 CYS:36:4 CYS:39:4
2010<br>2	3j0y	CYS:16:2 CYS:18:2 CYS:37:2 CYS:40:2
2010<br>2	2xux	CYS:16:4 CYS:18:4 CYS:36:4 CYS:39:4
2010<br>2	2hgq	CYS:16:3 CYS:18:3 CYS:36:3 CYS:39:3
2010<br>2	2y15	CYS:16:4 CYS:18:4 CYS:36:4 CYS:39:4
2010<br>9	3izs	CYS:44:i CYS:47:i CYS:81:i CYS:84:i
2013<br>9	1sx0	CYS:8:A CYS:10:A CYS:19:A HIS:20:A
2046<br>4	2wp8	CYS:47:J CYS:52:J CYS:55:J HIS:184:J
2046<br>4	5g06	CYS:47:J CYS:52:J CYS:55:J HIS:184:J
2153<br>3	5k0y	CYS:23:k CYS:26:k CYS:74:k CYS:77:k
2153<br>3	3j3a	CYS:23:a CYS:26:a CYS:74:a CYS:77:a
2153<br>3	4d5l	CYS:23:a CYS:26:a CYS:74:a CYS:77:a
2153<br>3	4d61	CYS:23:a CYS:26:a CYS:74:a CYS:77:a
2153<br>3	4kzz	CYS:23:a CYS:26:a CYS:74:a CYS:77:a
2153<br>3	4kzx	CYS:23:a CYS:26:a CYS:74:a CYS:77:a
2153<br>3	4kzy	CYS:23:a CYS:26:a CYS:74:a CYS:77:a
2153<br>3	3j38	CYS:23:a CYS:26:a CYS:74:a CYS:77:a

21533	3zey	CYS:25:V CYS:28:V CYS:78:V CYS:81:V
21553	1r6c	HIS:20:X HIS:22:X GLU:63:X
21553	1mg9	HIS:20:B HIS:22:B GLU:63:B
21553	1mbu	HIS:20:B HIS:22:B GLU:63:B
21553	1mbv	HIS:20:A HIS:22:A GLU:63:A
21553	1lzw	HIS:111:B HIS:113:B GLU:154:B
21553	1mbu	HIS:20:A HIS:22:A GLU:63:A
21553	1k6k	HIS:20:A HIS:22:A GLU:63:A
21615	2ps3	GLU:59:A HIS:137:A HIS:143:A HIS:207:A
21650	1t39	CYS:5:A CYS:24:A HIS:29:A HIS:85:A
21678	1pwv	HIS:686:A HIS:690:A GLU:735:A
21678	1pwv	HIS:686:B HIS:690:B GLU:735:B
21678	1jky	HIS:686:A HIS:690:A GLU:735:A
21933	2k8f	HIS:70:A CYS:74:A CYS:79:A CYS:84:A
21933	2mzd	HIS:70:A CYS:74:A CYS:79:A CYS:84:A
21933	2mh0	HIS:1792:B CYS:1796:B CYS:1801:B CYS:1806:B
21934	2mh0	HIS:1744:B CYS:1748:B CYS:1753:B CYS:1758:B
21934	2mzd	HIS:22:A CYS:26:A CYS:31:A CYS:36:A
21934	2k8f	HIS:22:A CYS:26:A CYS:31:A CYS:36:A
21936	2mh0	HIS:1767:B CYS:1771:B CYS:1779:B CYS:1782:B
21936	2k8f	HIS:45:A CYS:49:A CYS:57:A CYS:60:A
21936	2mzd	HIS:45:A CYS:49:A CYS:57:A CYS:60:A
22022	5h47	CYS:244:L ASP:246:L HIS:252:L
22022	4uou	CYS:244:A ASP:246:A HIS:252:A
22022	5h47	CYS:244:F ASP:246:F HIS:252:F

22022	5h47	CYS:244:E ASP:246:E HIS:252:E
22022	4aha	CYS:244:B ASP:246:B HIS:252:B
22022	5h47	CYS:244:H ASP:246:H HIS:252:H
22022	4aha	CYS:244:A ASP:246:A HIS:252:A
22022	5h47	CYS:244:G ASP:246:G HIS:252:G
22022	5h47	CYS:244:C ASP:246:C HIS:252:C
22022	4agt	CYS:244:B ASP:246:B HIS:252:B
22022	5eo8	CYS:244:A ASP:246:A HIS:252:A
22022	4uou	CYS:244:C ASP:246:C HIS:252:C
22022	5eo7	CYS:244:C ASP:246:C HIS:252:C
22022	4agt	CYS:244:A ASP:246:A HIS:252:A
22022	5eo7	CYS:244:B ASP:246:B HIS:252:B
22022	5h47	CYS:244:D ASP:246:D HIS:252:D
22022	5h47	CYS:244:K ASP:246:K HIS:252:K
22022	4c1y	CYS:244:D ASP:246:D HIS:252:D
22022	5eo7	CYS:244:A ASP:246:A HIS:252:A
22022	5h47	CYS:244:J ASP:246:J HIS:252:J
22022	4uou	CYS:244:B ASP:246:B HIS:252:B
22022	4c1y	CYS:244:C ASP:246:C HIS:252:C
22022	5h47	CYS:244:B ASP:246:B HIS:252:B
22022	4c1y	CYS:244:B ASP:246:B HIS:252:B
22022	5h47	CYS:244:I ASP:246:I HIS:252:I
22022	5h47	CYS:244:A ASP:246:A HIS:252:A
22022	4uou	CYS:244:D ASP:246:D HIS:252:D
22119	3j1n	CYS:7:I CYS:10:I CYS:29:I CYS:32:I

22119	5c44	CYS:7:I CYS:10:I CYS:29:I CYS:32:I
22119	5u0s	CYS:7:i CYS:10:i CYS:29:i CYS:32:i
22313	4o8y	HIS:109:B HIS:111:B ASP:122:B
22313	5gjq	HIS:113:V HIS:115:V ASP:126:V
22313	5mpe	HIS:109:V HIS:111:V ASP:122:V
22313	4ocn	HIS:109:B HIS:111:B ASP:122:B
22313	5a5b	HIS:109:V HIS:111:V ASP:122:V
22313	4ocn	HIS:109:E HIS:111:E ASP:122:E
22313	4cr3	HIS:109:V HIS:111:V ASP:122:V
22313	4cr2	HIS:109:V HIS:111:V ASP:122:V
22313	5mpd	HIS:109:V HIS:111:V ASP:122:V
22313	4cr4	HIS:109:V HIS:111:V ASP:122:V
22386	2qvp	HIS:74:B GLU:77:B HIS:168:B
22386	2qvp	HIS:74:A GLU:77:A HIS:168:A
22386	2qvp	HIS:74:C GLU:77:C HIS:168:C
22621	3o2z	CYS:21:S CYS:24:S CYS:39:S CYS:42:S
22621	3izb	CYS:21:N CYS:24:N CYS:39:N CYS:42:N
22799	4fys	HIS:388:A HIS:392:A GLU:411:A
23247	1mhd	CYS:64:B CYS:109:B CYS:121:B HIS:126:B
23247	1mhd	CYS:64:A CYS:109:A CYS:121:A HIS:126:A
23257	4bxj	HIS:25:A HIS:146:A ASP:156:A
23257	4bxj	HIS:25:B HIS:146:B ASP:156:B
23257	4bj4	HIS:42:B HIS:153:B ASP:163:B
23257	4bxe	HIS:25:A HIS:146:A ASP:156:A
23257	2y2c	HIS:34:B HIS:154:B ASP:164:B

2325<br>7	4bxe	HIS:25:B HIS:146:B ASP:156:B
2325<br>7	3d2y	HIS:35:A HIS:151:A ASP:161:A
2325<br>7	4bj4	HIS:42:A HIS:153:A ASP:163:A
2325<br>7	2y2c	HIS:34:C HIS:154:C ASP:164:C
2325<br>7	2y2c	HIS:34:A HIS:154:A ASP:164:A
2325<br>7	4zxm	HIS:32:A HIS:141:A CYS:149:A
2326<br>9	4m6h	HIS:35:A GLU:70:A HIS:125:A
2330<br>6	2ykr	CYS:297:W CYS:302:W HIS:304:W<br>CYS:310:W
2341<br>2	1ilw	ASP:52:A HIS:54:A HIS:71:A
2374<br>1	1gau	CYS:7:A CYS:10:A CYS:28:A CYS:31:A
2387<br>0	4jrp	ASP:15:B GLU:17:B ASP:186:B
2387<br>0	4js4	ASP:15:B GLU:17:B ASP:186:B
2387<br>0	4js5	ASP:15:B GLU:17:B ASP:186:B
2387<br>0	4jrq	ASP:15:B GLU:17:B ASP:186:B
2387<br>0	4hcb	ASP:15:B GLU:17:B ASP:186:B
2387<br>0	4js5	ASP:15:A GLU:17:A ASP:186:A
2387<br>0	4jrq	ASP:15:A GLU:17:A ASP:186:A
2387<br>0	4js4	ASP:15:A GLU:17:A ASP:186:A
2387<br>0	4hcb	ASP:15:A GLU:17:A ASP:186:A
2392<br>7	3j39	CYS:12:o CYS:15:o CYS:72:o CYS:75:o
2392<br>7	3j3b	CYS:12:o CYS:15:o CYS:72:o CYS:77:o
2392<br>7	2zkr	CYS:12:4 CYS:15:4 CYS:72:4 CYS:77:4
2392<br>7	3zf7	CYS:12:t CYS:17:t CYS:74:t CYS:77:t
2408<br>8	3j1n	CYS:86:C CYS:88:C CYS:92:C CYS:95:C
2408<br>8	5c44	CYS:86:C CYS:88:C CYS:92:C CYS:95:C

2408 8	5u0s	CYS:90:c CYS:92:c CYS:96:c CYS:99:c
2416 8	3zf7	CYS:20:s CYS:23:s CYS:34:s CYS:39:s
2440 8	1x68	CYS:38:A CYS:41:A CYS:59:A CYS:62:A
2448 2	1d8e	ASP:297:B CYS:299:B HIS:362:B
2453 9	1vow	CYS:11:6 CYS:14:6 CYS:27:6 HIS:32:6
2453 9	3e1d	CYS:11:X CYS:14:X CYS:27:X HIS:33:X
2453 9	3izu	CYS:11:g CYS:14:g CYS:27:g HIS:33:g
2453 9	3j19	CYS:11:4 CYS:14:4 CYS:27:4 HIS:33:4
2453 9	3j37	CYS:11:9 CYS:14:9 CYS:27:9 HIS:33:9
2453 9	3j5l	CYS:11:4 CYS:14:4 CYS:27:4 HIS:33:4
2453 9	3uos	CYS:11:8 CYS:14:8 CYS:27:8 HIS:33:8
2453 9	1vp0	CYS:11:6 CYS:14:6 CYS:27:6 HIS:32:6
2453 9	3fik	CYS:11:4 CYS:14:4 CYS:27:4 HIS:33:4
2453 9	2zjr	CYS:11:4 CYS:14:4 CYS:27:4 HIS:32:4
2453 9	3j8g	CYS:11:8 CYS:14:8 CYS:27:8 HIS:33:8
2453 9	3izt	CYS:11:g CYS:14:g CYS:27:g HIS:33:g
2453 9	2b9n	CYS:11:9 CYS:14:9 CYS:27:9 HIS:32:9
2453 9	4io9	CYS:11:4 CYS:14:4 CYS:27:4 HIS:32:4
2453 9	3j0y	CYS:11:7 CYS:14:7 CYS:27:7 HIS:33:7
2453 9	2b66	CYS:11:9 CYS:14:9 CYS:27:9 HIS:32:9
2453 9	3e1b	CYS:11:X CYS:14:X CYS:27:X HIS:33:X
2453 9	2b9p	CYS:11:9 CYS:14:9 CYS:27:9 HIS:32:9
2453 9	4ioa	CYS:11:4 CYS:14:4 CYS:27:4 HIS:32:4
2453 9	2wwq	CYS:11:8 CYS:14:8 CYS:27:8 HIS:33:8
2453 9	3cf5	CYS:11:4 CYS:14:4 CYS:27:4 HIS:32:4

2453 9	1vou	CYS:11:6 CYS:14:6 CYS:27:6 HIS:32:6
2453 9	1yl3	CYS:11:9 CYS:14:9 CYS:27:9 HIS:32:9
2453 9	1vor	CYS:11:6 CYS:14:6 CYS:27:6 HIS:32:6
2453 9	4csu	CYS:11:8 CYS:14:8 CYS:27:8 HIS:33:8
2453 9	3j12	CYS:11:7 CYS:14:7 CYS:27:7 HIS:33:7
2453 9	3j01	CYS:11:4 CYS:14:4 CYS:27:4 HIS:33:4
2453 9	1voy	CYS:11:6 CYS:14:6 CYS:27:6 HIS:32:6
2453 9	3j14	CYS:11:7 CYS:14:7 CYS:27:7 HIS:33:7
2453 9	3kcr	CYS:11:4 CYS:14:4 CYS:27:4 HIS:33:4
2453 9	5ady	CYS:11:4 CYS:14:4 CYS:27:4 HIS:33:4
2453 9	4ioc	CYS:11:4 CYS:14:4 CYS:27:4 HIS:32:4
2453 9	3j0t	CYS:11:7 CYS:14:7 CYS:27:7 HIS:33:7
2453 9	3j7z	CYS:11:4 CYS:14:4 CYS:27:4 HIS:33:4
2453 9	2zjq	CYS:11:4 CYS:14:4 CYS:27:4 HIS:32:4
2453 9	3j0w	CYS:11:7 CYS:14:7 CYS:27:7 HIS:33:7
2453 9	3j11	CYS:11:7 CYS:14:7 CYS:27:7 HIS:33:7
2463 3	3uzn	CYS:13:6 CYS:16:6 CYS:40:6 CYS:43:6
2463 3	5mlc	CYS:15:3 CYS:18:3 CYS:52:3 CYS:55:3
2463 3	3i8i	CYS:13:6 CYS:16:6 CYS:40:6 CYS:43:6
2463 3	4kd2	CYS:13:6 CYS:16:6 CYS:40:6 CYS:43:6
2463 3	2y13	CYS:13:6 CYS:16:6 CYS:40:6 CYS:43:6
2463 3	2v49	CYS:13:6 CYS:16:6 CYS:40:6 CYS:43:6
2463 3	2xqe	CYS:13:6 CYS:16:6 CYS:40:6 CYS:43:6
2463 3	4k0q	CYS:13:6 CYS:16:6 CYS:40:6 CYS:43:6
2463 3	3uye	CYS:13:6 CYS:16:6 CYS:40:6 CYS:43:6

2463 3	3tve	CYS:13:6 CYS:16:6 CYS:40:6 CYS:43:6
2463 3	3i9e	CYS:13:6 CYS:16:6 CYS:40:6 CYS:43:6
2463 3	4kbw	CYS:13:6 CYS:16:6 CYS:40:6 CYS:43:6
2463 3	4kdk	CYS:13:6 CYS:16:6 CYS:40:6 CYS:43:6
2463 3	4kd9	CYS:13:6 CYS:16:6 CYS:40:6 CYS:43:6
2463 3	2wdn	CYS:13:6 CYS:16:6 CYS:40:6 CYS:43:6
2463 3	3uzk	CYS:13:6 CYS:16:6 CYS:40:6 CYS:43:6
2463 3	4kcz	CYS:13:6 CYS:16:6 CYS:40:6 CYS:43:6
2463 3	2wrr	CYS:13:6 CYS:16:6 CYS:40:6 CYS:43:6
2463 3	3uz9	CYS:13:6 CYS:16:6 CYS:40:6 CYS:43:6
2463 3	2wdj	CYS:13:6 CYS:16:6 CYS:40:6 CYS:43:6
2463 3	4ejc	CYS:13:6 CYS:16:6 CYS:40:6 CYS:43:6
2463 3	4g5l	CYS:13:6 CYS:16:6 CYS:40:6 CYS:43:6
2463 3	4ejb	CYS:13:6 CYS:16:6 CYS:40:6 CYS:43:6
2463 3	3tvh	CYS:13:6 CYS:16:6 CYS:40:6 CYS:43:6
2463 3	2hgu	CYS:13:5 CYS:16:5 CYS:40:5 CYS:43:5
2463 3	2wrl	CYS:13:6 CYS:16:6 CYS:40:6 CYS:43:6
2463 3	2hgj	CYS:13:5 CYS:16:5 CYS:40:5 CYS:43:5
2463 3	2hgq	CYS:13:5 CYS:16:5 CYS:40:5 CYS:43:5
2463 3	3uzf	CYS:13:6 CYS:16:6 CYS:40:6 CYS:43:6
2463 3	2x9u	CYS:13:6 CYS:16:6 CYS:40:6 CYS:43:6
2463 3	2j01	CYS:13:6 CYS:16:6 CYS:40:6 CYS:43:6
2463 3	4g5n	CYS:13:6 CYS:16:6 CYS:40:6 CYS:43:6
2463 3	4kfl	CYS:13:6 CYS:16:6 CYS:40:6 CYS:43:6
2463 3	4l6l	CYS:13:3 CYS:16:3 CYS:40:3 CYS:43:3

2463<br>3	2wdi	CYS:13:6 CYS:16:6 CYS:40:6 CYS:43:6
2463<br>3	2wdl	CYS:13:6 CYS:16:6 CYS:40:6 CYS:43:6
2463<br>3	3uzh	CYS:13:6 CYS:16:6 CYS:40:6 CYS:43:6
2463<br>3	3i9c	CYS:13:6 CYS:16:6 CYS:40:6 CYS:43:6
2463<br>3	2x9s	CYS:13:6 CYS:16:6 CYS:40:6 CYS:43:6
2463<br>3	3uz2	CYS:13:6 CYS:16:6 CYS:40:6 CYS:43:6
2463<br>3	4kdb	CYS:13:6 CYS:16:6 CYS:40:6 CYS:43:6
2463<br>3	4k0m	CYS:13:6 CYS:16:6 CYS:40:6 CYS:43:6
2463<br>3	2xtg	CYS:13:6 CYS:16:6 CYS:40:6 CYS:43:6
2463<br>3	2y0v	CYS:13:6 CYS:16:6 CYS:40:6 CYS:43:6
2463<br>3	4abs	CYS:13:6 CYS:16:6 CYS:40:6 CYS:43:6
2463<br>3	3zvp	CYS:13:6 CYS:16:6 CYS:40:6 CYS:43:6
2463<br>3	3zne	CYS:13:6 CYS:16:6 CYS:40:6 CYS:43:6
2463<br>3	2wh4	CYS:13:6 CYS:16:6 CYS:40:6 CYS:43:6
2463<br>3	3pyv	CYS:13:3 CYS:16:3 CYS:40:3 CYS:43:3
2463<br>3	3uz8	CYS:13:6 CYS:16:6 CYS:40:6 CYS:43:6
2463<br>3	3i8f	CYS:13:6 CYS:16:6 CYS:40:6 CYS:43:6
2463<br>3	4kfi	CYS:13:6 CYS:16:6 CYS:40:6 CYS:43:6
2463<br>3	2y17	CYS:13:6 CYS:16:6 CYS:40:6 CYS:43:6
2463<br>3	4kdh	CYS:13:6 CYS:16:6 CYS:40:6 CYS:43:6
2463<br>3	2y19	CYS:13:6 CYS:16:6 CYS:40:6 CYS:43:6
2463<br>3	2xg0	CYS:13:6 CYS:16:6 CYS:40:6 CYS:43:6
2463<br>3	2wh2	CYS:13:6 CYS:16:6 CYS:40:6 CYS:43:6
2463<br>3	2xg2	CYS:13:6 CYS:16:6 CYS:40:6 CYS:43:6
2463<br>3	3zn9	CYS:13:6 CYS:16:6 CYS:40:6 CYS:43:6

24633	2y0z	CYS:13:6 CYS:16:6 CYS:40:6 CYS:43:6
24633	2j03	CYS:13:6 CYS:16:6 CYS:40:6 CYS:43:6
24633	3v6x	CYS:13:6 CYS:16:6 CYS:40:6 CYS:43:6
24633	4kbu	CYS:13:6 CYS:16:6 CYS:40:6 CYS:43:6
24633	3uz1	CYS:13:6 CYS:16:6 CYS:40:6 CYS:43:6
24633	3v6w	CYS:13:6 CYS:16:6 CYS:40:6 CYS:43:6
24633	2y15	CYS:13:6 CYS:16:6 CYS:40:6 CYS:43:6
24633	2wro	CYS:13:6 CYS:16:6 CYS:40:6 CYS:43:6
24633	3fin	CYS:13:6 CYS:16:6 CYS:40:6 CYS:43:6
24633	2y11	CYS:13:6 CYS:16:6 CYS:40:6 CYS:43:6
24633	4g5u	CYS:13:6 CYS:16:6 CYS:40:6 CYS:43:6
24633	3uxq	CYS:13:6 CYS:16:6 CYS:40:6 CYS:43:6
24633	2wrj	CYS:13:6 CYS:16:6 CYS:40:6 CYS:43:6
24633	4b8g	CYS:13:6 CYS:16:6 CYS:40:6 CYS:43:6
24633	3uyg	CYS:13:6 CYS:16:6 CYS:40:6 CYS:43:6
24633	2v47	CYS:13:6 CYS:16:6 CYS:40:6 CYS:43:6
24633	4g5w	CYS:13:6 CYS:16:6 CYS:40:6 CYS:43:6
24633	3uxr	CYS:13:6 CYS:16:6 CYS:40:6 CYS:43:6
24633	4b8i	CYS:13:6 CYS:16:6 CYS:40:6 CYS:43:6
24633	3bbo	CYS:15:3 CYS:18:3 CYS:52:3 CYS:55:3
24633	2y0x	CYS:13:6 CYS:16:6 CYS:40:6 CYS:43:6
24633	4jux	CYS:13:6 CYS:16:6 CYS:40:6 CYS:43:6
24633	2xux	CYS:13:6 CYS:16:6 CYS:40:6 CYS:43:6
24898	2f3n	GLU:21:A HIS:22:A HIS:54:A
24898	2f3n	GLU:21:C HIS:22:C HIS:54:C

24898	2f3n	GLU:21:B HIS:22:B HIS:54:B
24903	1jfw	CYS:22:A HIS:33:A CYS:34:A CYS:37:A
24903	1tbc	CYS:22:A HIS:33:A CYS:34:A CYS:37:A
24903	1k5k	CYS:22:A HIS:33:A CYS:34:A CYS:37:A
24903	1tiv	CYS:22:A HIS:33:A CYS:34:A CYS:37:A
26086	1rik	CYS:5:A CYS:8:A HIS:21:A HIS:25:A
26086	1njq	CYS:8:A CYS:11:A HIS:24:A HIS:28:A
26139	4umm	CYS:183:E CYS:189:E CYS:199:E CYS:202:E
26139	4umm	CYS:149:A CYS:155:A CYS:165:A CYS:168:A
26140	4umm	CYS:113:A CYS:116:A CYS:130:A CYS:133:A
26140	4umm	CYS:147:E CYS:150:E CYS:164:E CYS:167:E
26395	1ivs	CYS:176:B CYS:179:B CYS:344:B CYS:347:B
26395	1ivs	CYS:176:A CYS:179:A CYS:344:A CYS:347:A
26397	1ivs	CYS:417:A CYS:438:A CYS:441:A
26397	1ivs	CYS:417:B CYS:438:B CYS:441:B
26865	5dsq	HIS:94:A HIS:96:A HIS:119:A
26865	4kp8	HIS:91:A HIS:93:A HIS:117:A
26865	4kp8	HIS:91:B HIS:93:B HIS:117:B
26865	4kp5	HIS:91:D HIS:93:D HIS:117:D
26865	5dsp	HIS:94:A HIS:96:A HIS:119:A
26865	4knm	HIS:96:B HIS:98:B HIS:121:B
26865	1fqn	HIS:94:A HIS:96:A HIS:119:A
26865	4kp8	HIS:91:D HIS:93:D HIS:117:D
26865	1fsn	HIS:94:B HIS:96:B HIS:119:B
26865	4q9y	HIS:94:A HIS:96:A HIS:119:A

26865	3d93	HIS:94:A HIS:96:A HIS:119:A
26865	1fsn	HIS:94:A HIS:96:A HIS:119:A
26865	4kp5	HIS:91:B HIS:93:B HIS:117:B
26865	4knj	HIS:94:A HIS:96:A HIS:119:A
26865	4kp5	HIS:91:C HIS:93:C HIS:117:C
26865	4kp8	HIS:91:C HIS:93:C HIS:117:C
26865	4q81	HIS:94:A HIS:96:A HIS:119:A
26865	4kp5	HIS:91:A HIS:93:A HIS:117:A
26865	5dso	HIS:94:A HIS:96:A HIS:119:A
26865	5dsr	HIS:94:A HIS:96:A HIS:119:A
26865	1zsa	HIS:94:A HIS:96:A HIS:119:A
26865	2cbe	HIS:94:A HIS:96:A HIS:119:A
26865	4knm	HIS:96:A HIS:98:A HIS:121:A
34042	4p5u	GLU:91:A HIS:127:A HIS:152:A
34042	4pe8	GLU:91:A HIS:127:A HIS:152:A
34056	2fui	CYS:11:A CYS:13:A HIS:34:A CYS:37:A
34074	1ozt	HIS:48:K HIS:63:K HIS:120:K
34074	1ozt	HIS:48:J HIS:63:J HIS:120:J
34074	1ozt	HIS:48:G HIS:63:G HIS:120:G
34074	1ozt	HIS:48:L HIS:63:L HIS:120:L
34074	3k91	HIS:63:B HIS:80:B ASP:83:B
34074	1ozt	HIS:48:I HIS:63:I HIS:120:I
34074	1ozt	HIS:48:H HIS:63:H HIS:120:H
34325	5lcw	CYS:51:B HIS:53:B CYS:73:B CYS:76:B
35542	3t6j	HIS:450:A HIS:455:A GLU:508:A

35542	3t6b	HIS:450:A HIS:455:A GLU:508:A
35542	3t6b	HIS:450:B HIS:455:B GLU:508:B
35790	2bxk	HIS:67:A HIS:247:A ASP:249:A
35790	4k2c	HIS:67:A HIS:247:A ASP:249:A
35790	4l9k	HIS:67:A HIS:247:A ASP:249:A
35790	4iw1	HIS:67:A HIS:247:A ASP:249:A
35790	4hgk	HIS:67:A HIS:247:A ASP:249:A
35790	4lb9	HIS:67:A HIS:247:A ASP:249:A
35790	2vue	HIS:67:A HIS:247:A ASP:249:A
35790	5ghk	HIS:67:A HIS:247:A ASP:249:A
35790	1e7c	HIS:67:A HIS:247:A ASP:249:A
35790	1e7a	HIS:67:A HIS:247:A ASP:249:A
35790	2xvu	HIS:67:B HIS:247:B ASP:249:B
35790	2bxg	HIS:67:A HIS:247:A ASP:249:A
35790	4g04	HIS:67:A HIS:247:A ASP:249:A
35790	1bj5	HIS:67:A HIS:247:A ASP:249:A
35790	5id7	HIS:67:A HIS:247:A ASP:249:A
35790	4n0f	HIS:67:M HIS:247:M ASP:249:M
35790	3b9l	HIS:67:A HIS:247:A ASP:249:A
35790	3lu6	HIS:67:A HIS:247:A ASP:249:A
35790	2vuf	HIS:67:B HIS:247:B ASP:249:B
35790	4f5u	HIS:67:A HIS:246:A ASP:248:A
35790	4la0	HIS:67:B HIS:247:B ASP:249:B
35790	2bxe	HIS:67:A HIS:247:A ASP:249:A
35790	2i2z	HIS:67:A HIS:247:A ASP:249:A

3579<br>0	1e7h	HIS:67:A HIS:247:A ASP:249:A
3579<br>0	3lu8	HIS:67:B HIS:247:B ASP:249:B
3579<br>0	4lb2	HIS:67:A HIS:247:A ASP:249:A
3579<br>0	5id9	HIS:67:A HIS:246:A ASP:248:A
3579<br>0	2bxh	HIS:67:B HIS:247:B ASP:249:B
3579<br>0	4g03	HIS:67:B HIS:247:B ASP:249:B
3579<br>0	2bxb	HIS:67:A HIS:247:A ASP:249:A
3579<br>0	2bxb	HIS:67:B HIS:247:B ASP:249:B
3579<br>0	4z69	HIS:67:A HIS:247:A ASP:249:A
3579<br>0	4k71	HIS:67:A HIS:247:A ASP:249:A
3579<br>0	2vue	HIS:67:B HIS:247:B ASP:249:B
3579<br>0	1e7b	HIS:67:B HIS:247:B ASP:249:B
3579<br>0	1e7i	HIS:67:A HIS:247:A ASP:249:A
3579<br>0	2i30	HIS:67:A HIS:247:A ASP:249:A
3579<br>0	1ao6	HIS:67:A HIS:247:A ASP:249:A
3579<br>0	3sqj	HIS:67:B HIS:247:B ASP:249:B
3579<br>0	5dby	HIS:67:A HIS:246:A ASP:248:A
3579<br>0	1hk1	HIS:67:A HIS:247:A ASP:249:A
3579<br>0	2xvw	HIS:67:A HIS:247:A ASP:249:A
3579<br>0	4j2v	HIS:67:A HIS:246:A ASP:248:A
3579<br>0	2bxc	HIS:67:A HIS:247:A ASP:249:A
3579<br>0	2bxg	HIS:67:B HIS:247:B ASP:249:B
3579<br>0	1e7a	HIS:67:B HIS:247:B ASP:249:B
3579<br>0	1n5u	HIS:67:A HIS:247:A ASP:249:A
3579<br>0	3jqz	HIS:67:B HIS:247:B ASP:249:B

3579<br>0	4f5v	HIS:67:A HIS:247:A ASP:249:A
3579<br>0	1o9x	HIS:67:A HIS:247:A ASP:249:A
3579<br>0	3lu6	HIS:67:B HIS:247:B ASP:249:B
3579<br>0	4ot2	HIS:67:A HIS:246:A ASP:248:A
3579<br>0	2bxc	HIS:67:B HIS:247:B ASP:249:B
3579<br>0	2vuf	HIS:67:A HIS:247:A ASP:249:A
3579<br>0	4l9q	HIS:67:A HIS:247:A ASP:249:A
3579<br>0	2bxe	HIS:67:B HIS:247:B ASP:249:B
3579<br>0	4g03	HIS:67:A HIS:247:A ASP:249:A
3579<br>0	3cx9	HIS:67:A HIS:247:A ASP:249:A
3579<br>0	1e7b	HIS:67:A HIS:247:A ASP:249:A
3579<br>0	1.00E+78	HIS:67:A HIS:247:A ASP:249:A
3579<br>0	2bxl	HIS:67:A HIS:247:A ASP:249:A
3579<br>0	3lu8	HIS:67:A HIS:247:A ASP:249:A
3579<br>0	4la0	HIS:67:A HIS:247:A ASP:249:A
3579<br>0	2bxp	HIS:67:A HIS:247:A ASP:249:A
3579<br>0	3jry	HIS:67:A HIS:247:A ASP:249:A
3579<br>0	1ao6	HIS:67:B HIS:247:B ASP:249:B
3579<br>0	3uiv	HIS:67:A HIS:247:A ASP:249:A
3579<br>0	1hk4	HIS:67:A HIS:247:A ASP:249:A
3579<br>0	5fuo	HIS:67:A HIS:247:A ASP:249:A
3579<br>0	1h9z	HIS:67:A HIS:247:A ASP:249:A
3579<br>0	2bxi	HIS:67:A HIS:247:A ASP:249:A
3579<br>0	3lu7	HIS:67:A HIS:247:A ASP:249:A
3579<br>0	4n0u	HIS:67:D HIS:247:D ASP:249:D

3579<br>0	3jry	HIS:67:B HIS:247:B ASP:249:B
3579<br>0	4bke	HIS:67:A HIS:247:A ASP:249:A
3579<br>0	1.00E+78	HIS:67:B HIS:247:B ASP:249:B
3579<br>0	4zbr	HIS:67:A HIS:246:A ASP:248:A
3579<br>0	1e7g	HIS:67:A HIS:247:A ASP:249:A
3579<br>0	2bxh	HIS:67:A HIS:247:A ASP:249:A
3579<br>0	1hk5	HIS:67:A HIS:247:A ASP:249:A
3579<br>0	2bxd	HIS:67:A HIS:247:A ASP:249:A
3579<br>0	2bxf	HIS:67:A HIS:247:A ASP:249:A
3579<br>0	4l9q	HIS:67:B HIS:247:B ASP:249:B
3579<br>0	4or0	HIS:67:B HIS:246:B ASP:248:B
3579<br>0	2bxd	HIS:67:B HIS:247:B ASP:249:B
3579<br>0	1gnj	HIS:67:A HIS:247:A ASP:249:A
3579<br>0	4iw2	HIS:67:A HIS:247:A ASP:249:A
3579<br>0	2ydf	HIS:67:A HIS:247:A ASP:249:A
3579<br>0	1bm0	HIS:67:B HIS:247:B ASP:249:B
3579<br>0	3uiv	HIS:67:H HIS:247:H ASP:249:H
3579<br>0	2xvu	HIS:67:A HIS:247:A ASP:249:A
3579<br>0	1gni	HIS:67:A HIS:247:A ASP:249:A
3579<br>0	4jk4	HIS:67:A HIS:246:A ASP:248:A
3579<br>0	3tdl	HIS:67:A HIS:247:A ASP:249:A
3579<br>0	2bxm	HIS:67:A HIS:247:A ASP:249:A
3579<br>0	4g04	HIS:67:B HIS:247:B ASP:249:B
3579<br>0	3a73	HIS:67:B HIS:247:B ASP:249:B
3579<br>0	3b9m	HIS:67:A HIS:247:A ASP:249:A

3579<br>0	4f5s	HIS:67:A HIS:246:A ASP:248:A
3579<br>0	2ydf	HIS:67:B HIS:247:B ASP:249:B
3579<br>0	2bxq	HIS:67:A HIS:247:A ASP:249:A
3579<br>0	5ifo	HIS:67:A HIS:247:A ASP:249:A
3579<br>0	4emx	HIS:67:B HIS:247:B ASP:249:B
3579<br>0	4.00E+99	HIS:67:A HIS:247:A ASP:249:A
3579<br>0	1e7e	HIS:67:A HIS:247:A ASP:249:A
3579<br>0	4s1y	HIS:67:A HIS:247:A ASP:249:A
3579<br>0	3v08	HIS:67:A HIS:246:A ASP:248:A
3579<br>0	4l8u	HIS:67:A HIS:247:A ASP:249:A
3579<br>0	4hgm	HIS:67:B HIS:247:B ASP:249:B
3579<br>0	2bxf	HIS:67:B HIS:247:B ASP:249:B
3579<br>0	3jqz	HIS:67:A HIS:247:A ASP:249:A
3579<br>0	4k71	HIS:67:D HIS:247:D ASP:249:D
3579<br>0	4z69	HIS:67:I HIS:247:I ASP:249:I
3579<br>0	4n0f	HIS:67:J HIS:247:J ASP:249:J
3579<br>0	5id7	HIS:67:B HIS:247:B ASP:249:B
3579<br>0	2xvv	HIS:67:A HIS:247:A ASP:249:A
3579<br>0	5dqf	HIS:67:A HIS:246:A ASP:248:A
3579<br>0	2xvq	HIS:67:B HIS:247:B ASP:249:B
3579<br>0	4emx	HIS:67:A HIS:247:A ASP:249:A
3579<br>0	4luh	HIS:67:A HIS:246:A ASP:248:A
3579<br>0	2vdb	HIS:67:A HIS:247:A ASP:249:A
3579<br>0	1hk2	HIS:67:A HIS:247:A ASP:249:A
3579<br>0	2bxo	HIS:67:A HIS:247:A ASP:249:A

3579 0	4jk4	HIS:67:B HIS:246:B ASP:248:B
3579 0	2bxa	HIS:67:B HIS:247:B ASP:249:B
3579 0	4or0	HIS:67:A HIS:246:A ASP:248:A
3579 0	1bke	HIS:67:A HIS:247:A ASP:249:A
3579 0	2bx8	HIS:67:B HIS:247:B ASP:249:B
3579 0	2bxn	HIS:67:A HIS:247:A ASP:249:A
3579 0	2xvq	HIS:67:A HIS:247:A ASP:249:A
3579 0	3lu7	HIS:67:B HIS:247:B ASP:249:B
3579 0	5hoz	HIS:67:A HIS:246:A ASP:248:A
3579 0	2xsi	HIS:67:A HIS:247:A ASP:249:A
3579 0	3v09	HIS:67:A HIS:247:A ASP:249:A
3579 0	2xw1	HIS:67:A HIS:247:A ASP:249:A
3579 0	1bm0	HIS:67:A HIS:247:A ASP:249:A
3579 0	2bxa	HIS:67:A HIS:247:A ASP:249:A
3579 0	3a73	HIS:67:A HIS:247:A ASP:249:A
3579 0	4hgk	HIS:67:B HIS:247:B ASP:249:B
3579 0	4luf	HIS:67:A HIS:246:A ASP:248:A
3579 0	2bx8	HIS:67:A HIS:247:A ASP:249:A
3579 0	3sqj	HIS:67:A HIS:247:A ASP:249:A
3579 0	4lb2	HIS:67:B HIS:247:B ASP:249:B
3579 0	1e7f	HIS:67:A HIS:247:A ASP:249:A
3579 0	1tf0	HIS:67:A HIS:247:A ASP:249:A
3579 0	1hk3	HIS:67:A HIS:247:A ASP:249:A
3579 0	4zbq	HIS:67:A HIS:246:A ASP:248:A
3579 0	4k2c	HIS:67:B HIS:247:B ASP:249:B

35790	2xw0	HIS:67:B HIS:247:B ASP:249:B
35790	4n0f	HIS:67:D HIS:247:D ASP:249:D
35790	2xw0	HIS:67:A HIS:247:A ASP:249:A
35790	4po0	HIS:67:A HIS:247:A ASP:249:A
35790	4n0f	HIS:67:G HIS:247:G ASP:249:G
35790	1uor	HIS:67:A HIS:247:A ASP:249:A
35790	1ha2	HIS:67:A HIS:247:A ASP:249:A
35790	2xw1	HIS:67:B HIS:247:B ASP:249:B
35790	4f5s	HIS:67:B HIS:246:B ASP:248:B
35790	4l9k	HIS:67:B HIS:247:B ASP:249:B
35928	3wrf	GLU:338:A CYS:340:A CYS:417:A CYS:418:A
36033	4zbq	GLU:152:A HIS:156:A HIS:287:A
36033	4f5u	GLU:152:A HIS:156:A HIS:287:A
36033	5id9	GLU:152:A HIS:156:A HIS:287:A
36033	4zbr	GLU:152:A HIS:156:A HIS:287:A
36033	4j2v	GLU:152:A HIS:156:A HIS:287:A
36033	5dqf	GLU:152:A HIS:156:A HIS:287:A
36033	3v08	GLU:152:A HIS:156:A HIS:287:A
36033	5dby	GLU:152:A HIS:156:A HIS:287:A
36033	5hoz	GLU:152:A HIS:156:A HIS:287:A
36033	4ot2	GLU:152:A HIS:156:A HIS:287:A
36355	2kil	CYS:139:A HIS:161:A CYS:164:A CYS:172:A
36355	2kii	CYS:139:A HIS:161:A CYS:164:A CYS:172:A
36866	4bkn	HIS:73:B HIS:75:B ASP:172:B
36866	4bkn	HIS:73:A HIS:75:A ASP:172:A

36866	4cnu	HIS:73:B HIS:75:B ASP:172:B
36866	4cnu	HIS:73:A HIS:75:A ASP:172:A
37212	4rey	HIS:18:A HIS:20:A CYS:103:A
39119	5a88	HIS:186:D ASP:321:D HIS:325:D
39119	5a88	HIS:186:A ASP:321:A HIS:325:A
41410	2mmh	CYS:14:A HIS:16:A CYS:23:A CYS:26:A
41410	2mmk	CYS:14:A HIS:16:A CYS:23:A CYS:26:A
23079	2y17	CYS:11:9 CYS:14:9 CYS:27:9 HIS:32:9
74921	5mlc	CYS:11:6 CYS:14:6 CYS:27:6 HIS:32:6
32860	2y13	CYS:11:9 CYS:14:9 CYS:27:9 HIS:32:9
32860	1vsa	CYS:11:b CYS:14:b CYS:27:b HIS:32:b
32860	2wrl	CYS:11:9 CYS:14:9 CYS:27:9 HIS:32:9
32860	4kd2	CYS:11:9 CYS:14:9 CYS:27:9 HIS:32:9
32860	4ejc	CYS:11:9 CYS:14:9 CYS:27:9 HIS:32:9
32860	2yox	CYS:11:9 CYS:14:9 CYS:27:9 HIS:32:9'
32860	3v6x	CYS:11:9 CYS:14:9 CYS:27:9 HIS:32:9
32860	4kd9	CYS:11:9 CYS:14:9 CYS:27:9 HIS:32:9
32860	2wro	CYS:11:9 CYS:14:9 CYS:27:9 HIS:32:9
32860	2xqe	CYS:11:9 CYS:14:9 CYS:27:9 HIS:32:9
32860	2wdn	CYS:11:9 CYS:14:9 CYS:27:9 HIS:32:9
32860	2xtg	CYS:11:9 CYS:14:9 CYS:27:9 HIS:32:9
32860	4kbw	CYS:11:9 CYS:14:9 CYS:27:9 HIS:32:9
32860	2b9n	CYS:6:T CYS:9:T CYS:32:T CYS:36:T
32860	2y0z	CYS:11:9 CYS:14:9 CYS:27:9 HIS:32:9
32860	3zvp	CYS:11:9 CYS:14:9 CYS:27:9 HIS:32:9

32860	4abs	CYS:11:9 CYS:14:9 CYS:27:9 HIS:32:9
32860	4kdk	CYS:11:9 CYS:14:9 CYS:27:9 HIS:32:9
32860	2y15	CYS:11:9 CYS:14:9 CYS:27:9 HIS:32:9
32860	2b9p	CYS:6:T CYS:9:T CYS:32:T CYS:36:T
32860	1vsp	CYS:11:b CYS:14:b CYS:27:b HIS:32:b
32860	2hgj	CYS:11:8 CYS:14:8 CYS:27:8 HIS:32:8
32860	2wrr	CYS:11:9 CYS:14:9 CYS:27:9 HIS:32:9
32860	2hgq	CYS:11:8 CYS:14:8 CYS:27:8 HIS:32:8
32860	5lcw	CYS:23:B CYS:26:B CYS:34:B HIS:56:B HIS:58:B CYS:59:B
32860	4jux	CYS:11:9 CYS:14:9 CYS:27:9 HIS:32:9
32860	2hgu	CYS:11:8 CYS:14:8 CYS:27:8 HIS:32:8
32860	2y19	CYS:11:9 CYS:14:9 CYS:27:9 HIS:32:9
32860	4ejb	CYS:11:9 CYS:14:9 CYS:27:9 HIS:32:9
32860	2y0x	CYS:11:9 CYS:14:9 CYS:27:9 HIS:32:9
32860	2wdi	CYS:11:9 CYS:14:9 CYS:27:9 HIS:32:9
32860	4b8g	CYS:11:9 CYS:14:9 CYS:27:9 HIS:32:9
32860	2wh2	CYS:11:9 CYS:14:9 CYS:27:9 HIS:32:9
32860	3zn9	CYS:11:9 CYS:14:9 CYS:27:9 HIS:32:9
32860	2x9s	CYS:11:9 CYS:14:9 CYS:27:9 HIS:32:9
32860	2xux	CYS:11:9 CYS:14:9 CYS:27:9 HIS:32:9
32860	2b66	CYS:6:T CYS:9:T CYS:32:T CYS:36:T
32860	1yl3	CYS:6:R CYS:9:R CYS:32:R CYS:36:R
32860	5c44	CYS:75:I CYS:78:I CYS:103:I CYS:106:I
32860	4k0q	CYS:11:9 CYS:14:9 CYS:27:9 HIS:32:9
32860	4kcz	CYS:11:9 CYS:14:9 CYS:27:9 HIS:32:9

3286 0	2wdj	CYS:11:9 CYS:14:9 CYS:27:9 HIS:32:9
3286 0	2x9u	CYS:11:9 CYS:14:9 CYS:27:9 HIS:32:9
3286 0	2wdl	CYS:11:9 CYS:14:9 CYS:27:9 HIS:32:9
3286 0	4kdb	CYS:11:9 CYS:14:9 CYS:27:9 HIS:32:9
3286 0	2y0v	CYS:11:9 CYS:14:9 CYS:27:9 HIS:32:9
3286 0	3zne	CYS:11:9 CYS:14:9 CYS:27:9 HIS:32:9
3286 0	2wh4	CYS:11:9 CYS:14:9 CYS:27:9 HIS:32:9
3286 0	4kdh	CYS:11:9 CYS:14:9 CYS:27:9 HIS:32:9
3286 0	2y19	CYS:11:9 CYS:14:9 CYS:27:9 HIS:32:9
3286 0	2xg0	CYS:11:9 CYS:14:9 CYS:27:9 HIS:32:9
3286 0	2xg2	CYS:11:9 CYS:14:9 CYS:27:9 HIS:32:9
3286 0	4kbu	CYS:11:9 CYS:14:9 CYS:27:9 HIS:32:9
3286 0	3v6w	CYS:11:9 CYS:14:9 CYS:27:9 HIS:32:9
3286 0	3fin	CYS:11:9 CYS:14:9 CYS:27:9 HIS:32:9
3286 0	2y11	CYS:11:9 CYS:14:9 CYS:27:9 HIS:32:9
3286 0	2wrj	CYS:11:9 CYS:14:9 CYS:27:9 HIS:32:9
3286 0	4b8i	CYS:11:9 CYS:14:9 CYS:27:9 HIS:32:9
3286 0	3bbo	CYS:11:6 CYS:14:6 CYS:27:6 HIS:33:6

Table S2. Complete list of the selected proteins from the *S. cerevisiae* proteome that do not have homologous metalloproteins with a deposited 3D structure in the PDB, constituting the second benchmark.

**UniProt identifier**		
P25654	P28273	P32452
P35999	P38769	P40462
P53960	Q04638	P25618
P25625	P04801	P07259
P09932	P23060	P25297
P20967	P38138	P00812
P09880	P0CW40	P0CW41
P20604	P27810	P32598
P32775	P38130	P39965
Q05584	Q07747	Q08295
Q12090	Q12680	P38755
P38858	P39976	P42884
P42942	P47182	P54070
Q03262	Q12288	Q3E7A9
P32259	P32353	P36078
P36033	P38430	P38635
P36051	P39721	P40099
P40483	P40533	P53012
P53294	Q04235	Q01662
Q04847	P40825	Q05924
Q06667	Q08990	Q3E756
Q08634	Q08908	Q12393
P38693		

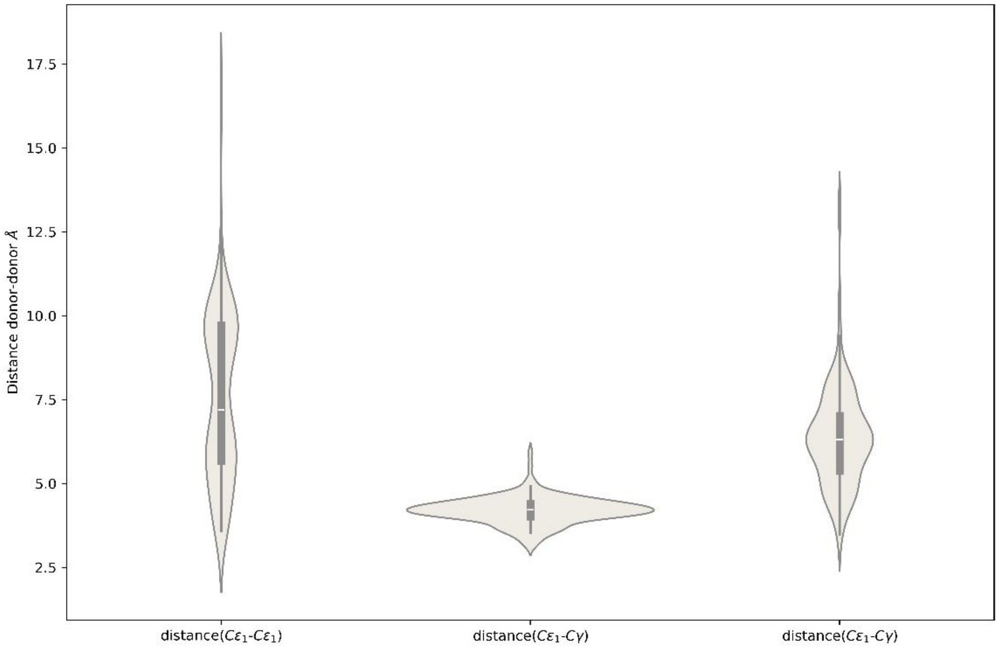

Figure S1. Violin plot for CLES 35790, composed of 158 sites.

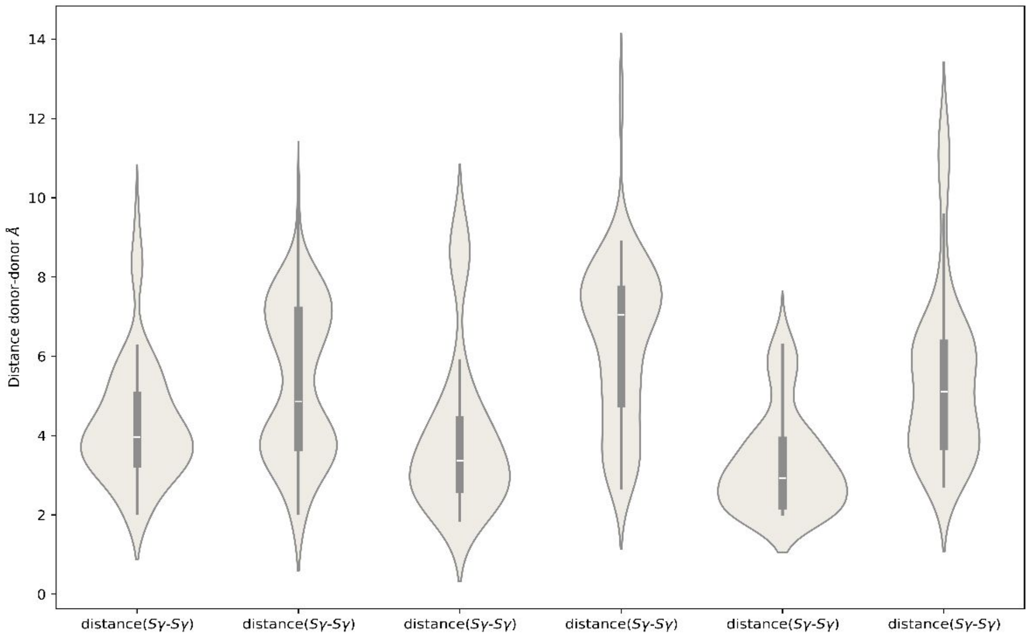

Figure S2. Violin plot for CLES 35790, composed of 83 sites.

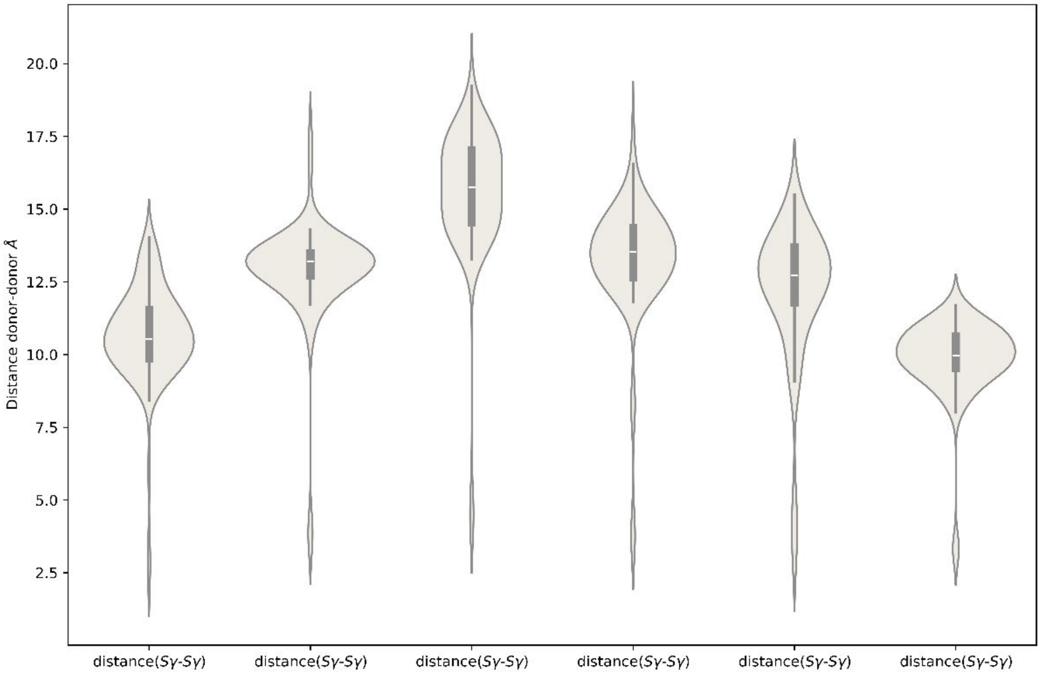

Figure S3. Violin plot for CLES 24633, composed of 82 sites.

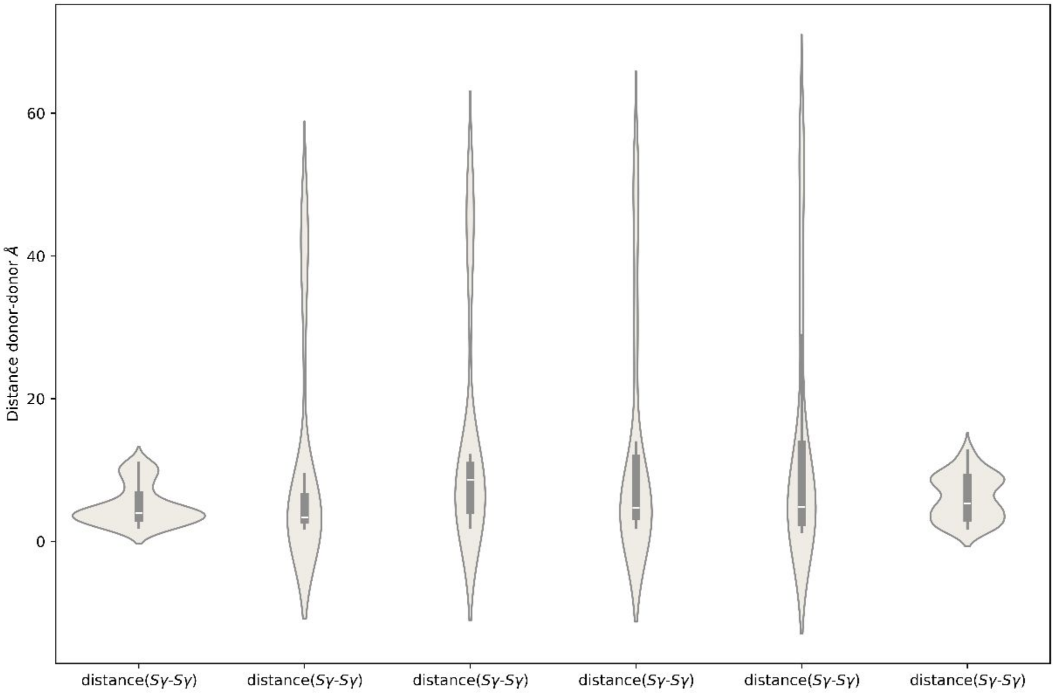

Figure S4. Violin plot for CLES 20102 composed of 75 sites.

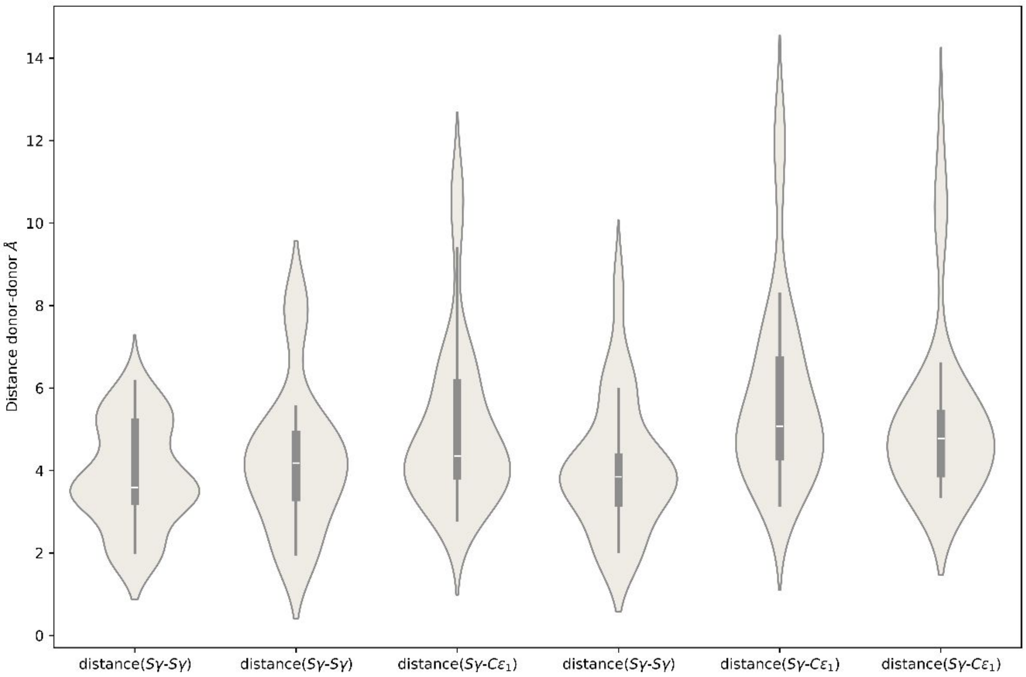

Figure S5. Violin plot for CLES 24539 composed of 37 sites.

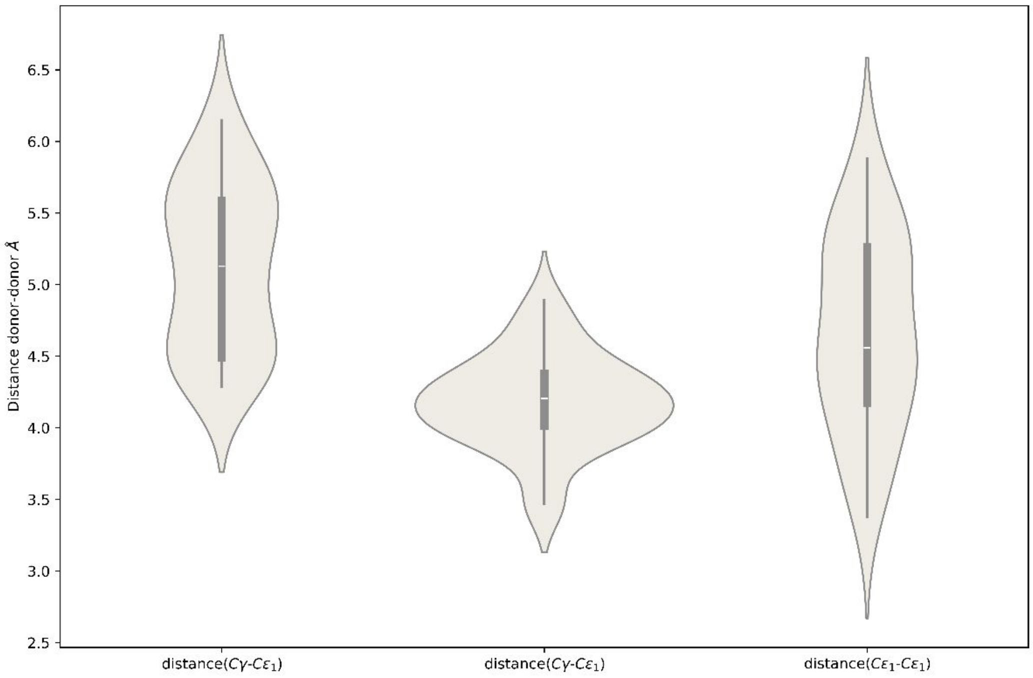

Figure S6. Violin plot for CLES 17339 composed of 27 sites.

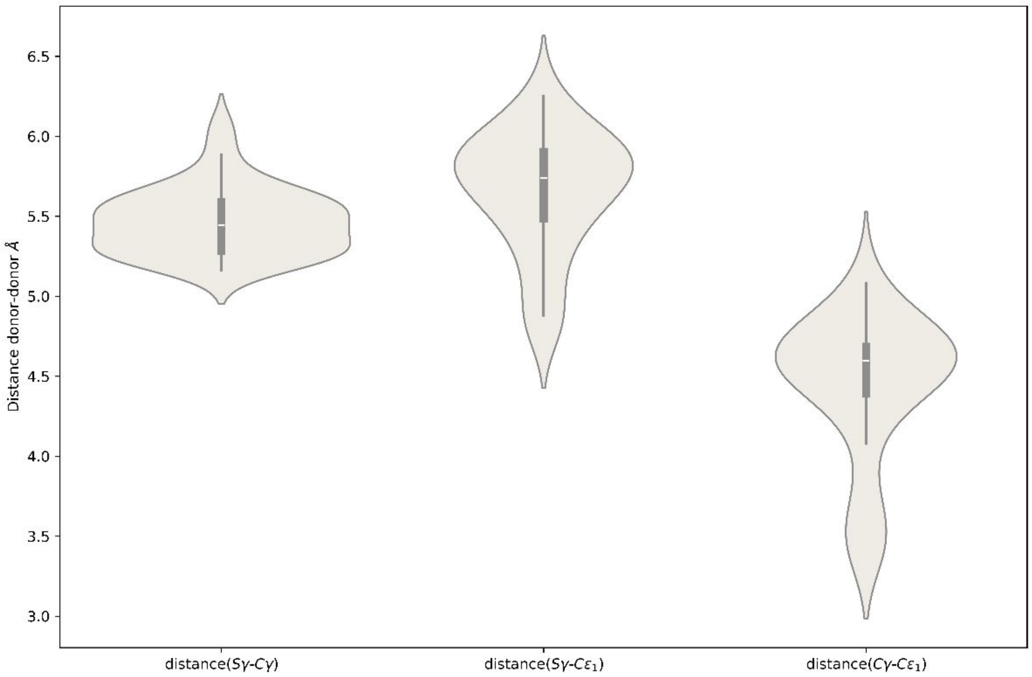

Figure S7. Violin plot for CLES 22022 composed of 27 sites.

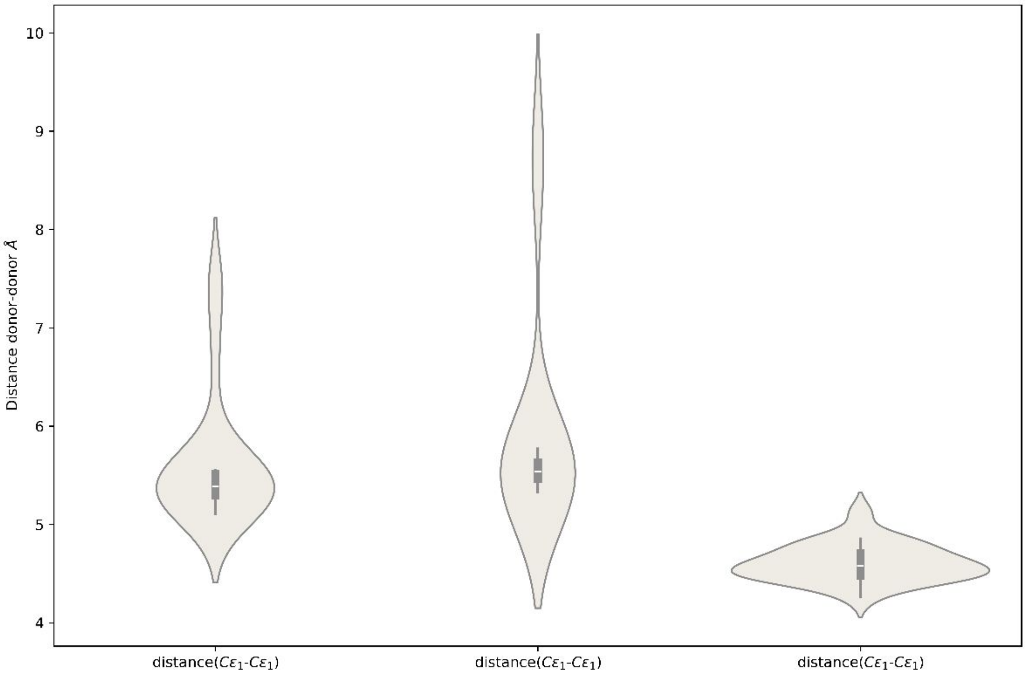

Figure S8. Violin plot for CLES 26865 composed of 23 sites.

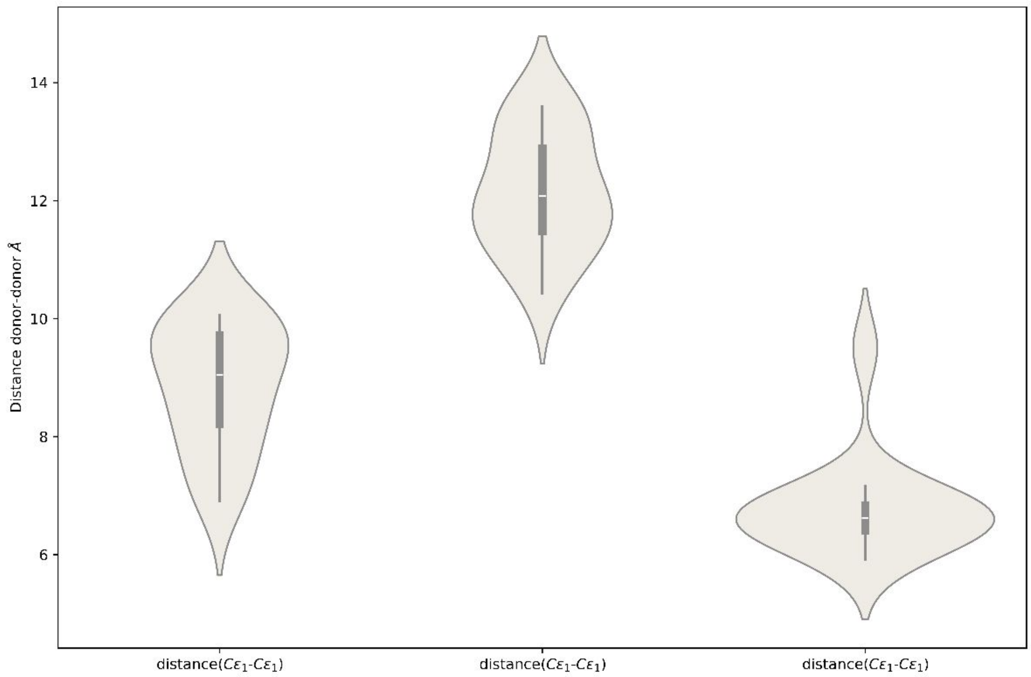

Figure S9. Violin plot for CLES 17910 composed of 14 sites.

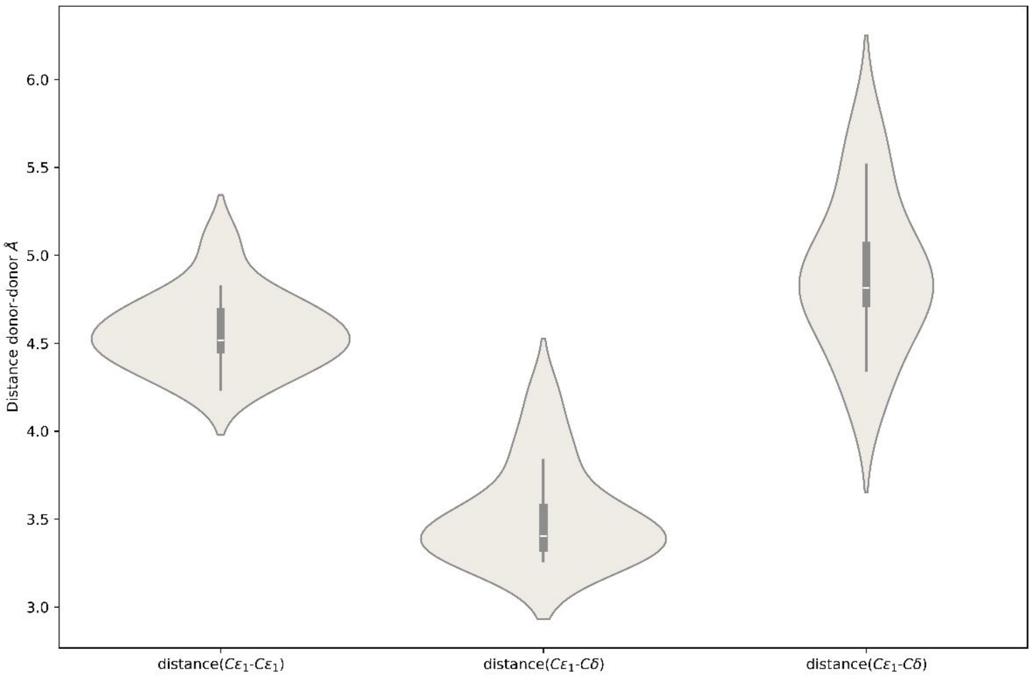

Figure S10. Violin plot for CLES 19742 composed of 14 sites.

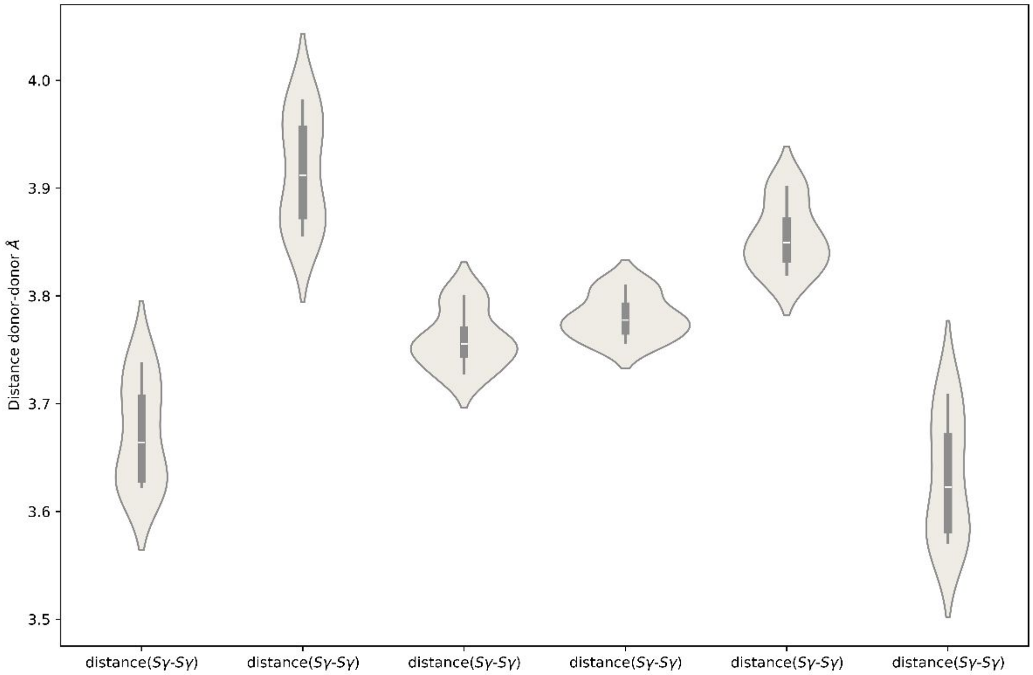

Figure S11. Violin plot for CLES 18235 composed of 12 sites.

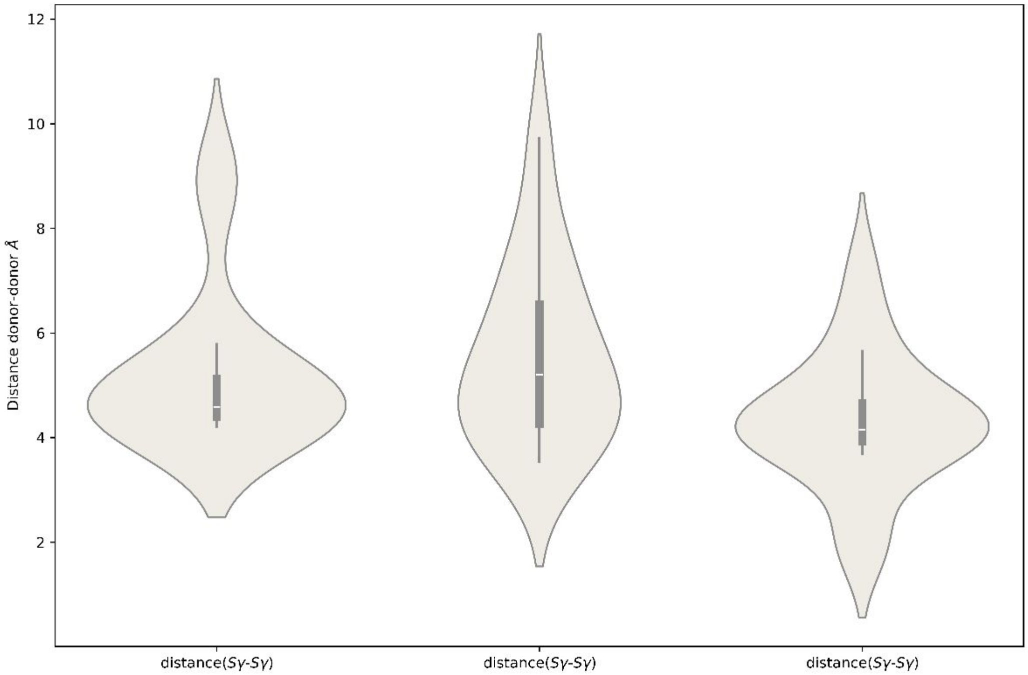

Figure S12. Violin plot for CLES 16944 composed of 11 sites.

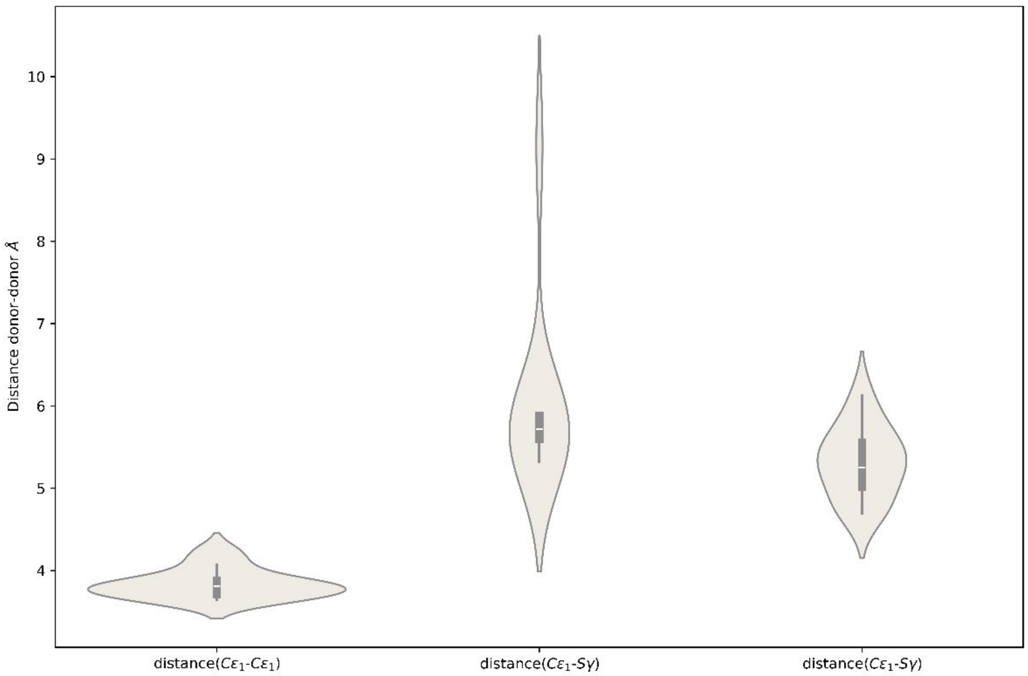

Figure S13. Violin plot for CLES 23257 composed of 11 sites.

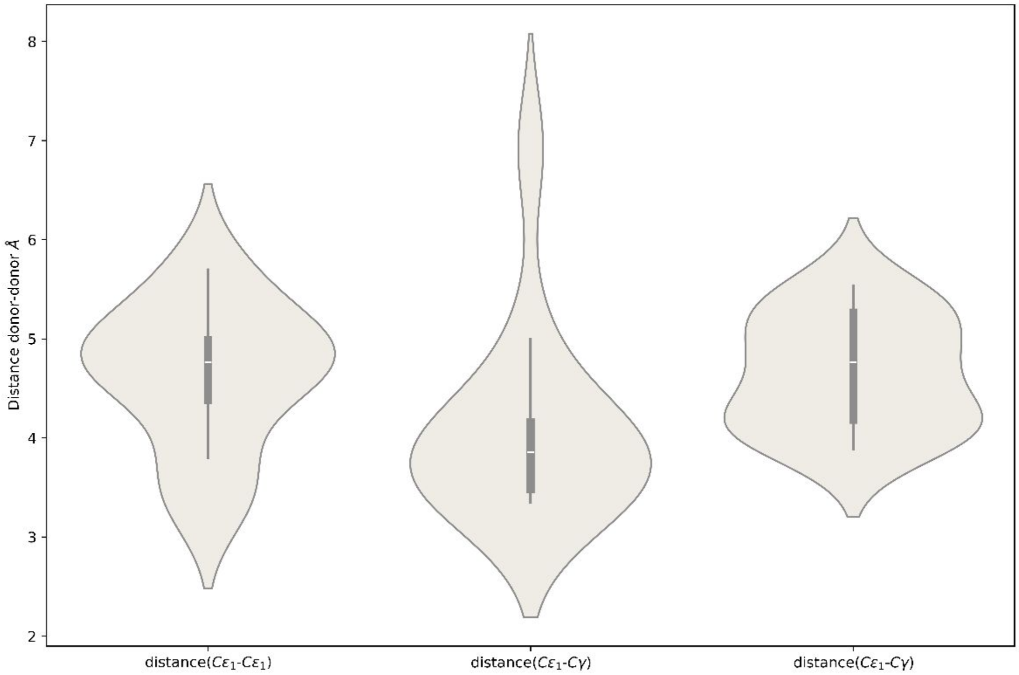

Figure S14. Violin plot for CLES 22313 composed of 10 sites.

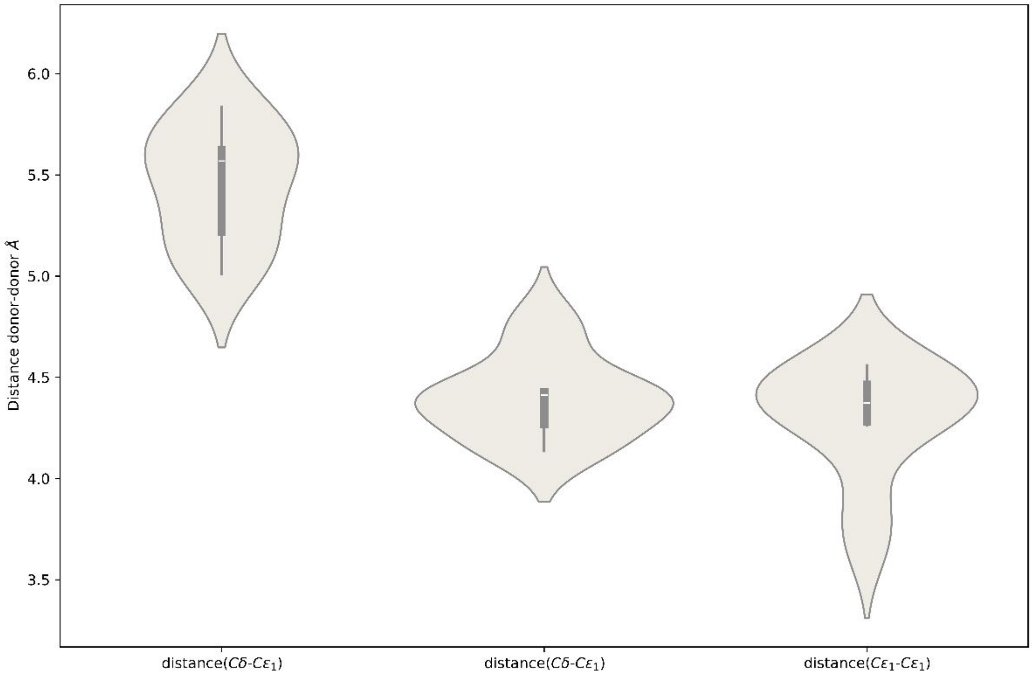

Figure S15. Violin plot for CLES 36033 composed of 10 sites.
